# Three cytosolic glutamine synthetase isoforms localized in different-order veins act together for N remobilization and seed filling in Arabidopsis

**DOI:** 10.1093/jxb/ery217

**Published:** 2018-06-05

**Authors:** Michael Moison, Anne Marmagne, Sylvie Dinant, Fabienne Soulay, Marianne Azzopardi, Jérémy Lothier, Sylvie Citerne, Halima Morin, Nicolas Legay, Fabien Chardon, Jean-Christophe Avice, Michèle Reisdorf-Cren, Céline Masclaux-Daubresse

**Affiliations:** 1 Institut Jean-Pierre Bourgin, INRA, AgroParisTech, CNRS, Université Paris-Saclay, Versailles, France; 2 Université de Versailles Saint Quentin en Yvelines, Université Paris Saclay, Versailles, France; 3 UCBN, INRA, UMR INRA-UBCN Ecophysiologie Végétale, Agronomie and Nutrition N.C.S., Université de Caen Normandie, Caen, France

**Keywords:** Ammonium, Asparagine synthetase, leaf senescence, ^15^N labelling, phloem, seed filling, yield

## Abstract

Glutamine synthetase (GS) is central for ammonium assimilation and consists of cytosolic (GS1) and chloroplastic (GS2) isoenzymes. During plant ageing, GS2 protein decreases due to chloroplast degradation, and GS1 activity increases to support glutamine biosynthesis and N remobilization from senescing leaves. The role of the different Arabidopsis GS1 isoforms in nitrogen remobilization was examined using ^15^N tracing experiments. Only the *gln1;1-gln1;2-gln1;3* triple-mutation affecting the three *GLN1;1*, *GLN1;2*, and *GLN1;3* genes significantly reduced N remobilization, total seed yield, individual seed weight, harvest index, and vegetative biomass. The triple-mutant accumulated a large amount of ammonium that could not be assimilated by GS1. Alternative ammonium assimilation through asparagine biosynthesis was increased and was related to higher *ASN2* asparagine synthetase transcript levels. The GS2 transcript, protein, and activity levels were also increased to compensate for the lack of GS1-related glutamine biosynthesis. Localization of the different *GLN1* genes showed that they were all expressed in the phloem companion cells but in veins of different order. Our results demonstrate that glutamine biosynthesis for N-remobilization occurs in veins of all orders (major and minor) in leaves, it is mainly catalysed by the three major GS1 isoforms (GLN1;1, GLN1;2, and GLN1;3), and it is alternatively supported by AS2 in the veins and GS2 in the mesophyll cells.

## Teaser

Glutamine biosynthesis for N-remobilization and seed filling in Arabidopsis is mainly catalysed by the three major GS1 isoforms, GLN1;1, GLN1;2, and GLN1;3, which are localized in different-order veins in the leaves.

## Introduction

Because their structures are more plastic than those of animals, plants regularly grow new organs to replace inefficient and senescing ones. In doing so, plants repeatedly remobilize nutrients from their senescing organs and tissues to facilitate the growth of the new organs, including the production and filling of seeds. Such nutrient remobilization is particularly important for plant regrowth after stress and also when mineral nutrients are scarce in the soil (Masclaux-Daubresse *et al.*, 2010). The mechanisms involved in the recycling and remobilization of nutrients are of great interest for improving plant performance and nitrogen use efficiency, and they have the potential to decrease the need for fertilzers and hence provide economies in crop production. Numerous physiological and omic studies examining senescing leaves and responses to nitrate limitation have identified many candidate mechanisms to focus on (Masclaux-Daubresse *et al.*, 2010; Tegeder and Masclaux-Daubresse, 2018). Among them, the cytosolic glutamine synthetases (GS1; EC 6.3.1.2) have been identified in many plant species (Guo *et al.*, 2004; Martin *et al.*, 2006; Diaz *et al.*, 2008; Avila-Ospina *et al.*, 2014).

Glutamine synthetase (GS) catalyses the biosynthesis of glutamine, which is one of the most abundant amino acids in phloem and xylem sap (Bernard and Habash, 2009; Masclaux-Daubresse *et al.*, 2010). GS1 activity is essential for the assimilation of ammonium in Arabidopsis (Lothier *et al.*, 2011; Guan *et al.*, 2016). During leaf senescence, ammonium is released via amino acid catabolism (Hildebrandt *et al.*, 2015). In many plant species, including Arabidopsis, the GS1 encoding genes are senescence-induced (Guo *et al.*, 2004; Diaz *et al.*, 2008; Avila-Ospina *et al.*, 2014). As chloroplasts collapse during leaf senescence, the chloroplastic GS2 glutamine synthetase isoenzyme is degraded and GS1 isoforms are induced, thus compensating for the decrease in GS2 and allowing the re-assimilation of ammonium to be catalysed (Diaz *et al.*, 2008; Orsel *et al.*, 2014; Avila-Ospina *et al.*, 2015). As such, GS1 has long been regarded as a central component in the nitrogen remobilization process. However, despite numerous studies and reviews reporting this fact, no demonstration of the role of GS1 in N-remobilization has ever been provided. The large number of GS1 isoforms found in all plant species has contributed to making their functional analysis difficult (Rodríguez *et al.*, 2011; Orsel *et al.*, 2014; Yamaya and Kusano, 2014; Avila-Ospina *et al.*, 2015). In the absence of mutants, quantification of nitrogen fluxes has not been able to demonstrate the role of GS1 in N remobilization.

In Arabidopsis, glutamine synthetase consists of one chloroplastic GS2 (GLN2) and five cytosolic GS1 isoforms (GLN1;1–GLN1;5) (Bernhard and Matile, 1994; Diaz *et al.*, 2008). It is accepted that the active GS1s are structured as homodecamers of GLN1 subunits (Unno *et al.*, 2006; Dragićević *et al.*, 2014). The different GS1 isoforms present different kinetic properties with respect to glutamate and ammonium: GLN1;1 and GLN1;4 have high affinity for ammonium while GLN1;2 and GLN1;3 have low affinity (Ishiyama *et al.*, 2004). The *GLN1* genes are differentially expressed in the roots and shoot and during leaf senescence (Ishiyama *et al.*, 2004; Lothier *et al.*, 2011; Avila-Ospina *et al.*, 2014). In a previous study, our group showed that mutations in the Arabidopsis *GLN1;2* gene affect plant growth but only under high-nitrate conditions and at the vegetative stage (Lothier *et al.*, 2011). Under high nitrate, the *gln1;2* mutants accumulate large amounts of ammonium, showing that GLN1;2 is essential for assimilation and detoxification of excess ammonium. This feature was confirmed by Guan *et al.* (2016), who used a hydroponic ammonium treatment system to show that GLN1;2 provides the main GS1 activity that plays a role in detoxification. Guan *et al.* (2015) showed that seed production and germination were impaired in the *gln1;2* single and the *gln1;2*-*gln1;1* double knock-out mutants. As a result of grafting experiments, Guan and Schjoerring (2016) proposed that the prominent role of GLN1;2 was in the shoot; however, in their studies, Konishi *et al.* (2017, 2018) showed that both GLN1;2 and GLN1;3 are essential for ammonium assimilation in the roots.

Focusing on the reproductive stage, ^15^N-tracing experiments performed by Lothier *et al.* (2011) showed that the *gln1;2* mutant was not affected in terms of N remobilization under either low- and high-nitrate conditions and it produced the same plant biomass, total seed yield, and seed C/N composition at harvest as the wild-type. The absence of defects in N remobilization was further confirmed by Guan and Schjoerring (2016). The aim of our current study was therefore to identify which *GLN1* isoforms in Arabidopsis are important for N remobilization and seed filling. We performed ^15^N pulse-chase labelling and tracing experiments on single, double, and triple knock-out mutants in order to quantify the fluxes of nitrogen remobilized to the seeds after flowering. Our results revealed that the three major isoforms GLN1;1, GLN1;2, and GLN1;3 compensate for each other and ensure efficient N remobilization for seed filling, although they were not localized in veins of the same order. Only the *gln1;1*-*gln1;2*-*gln1;3* triple-mutation significantly reduced N remobilization, thus demonstrating the essential complementary role of the three GLN1;1, GLN1;2, and GLN1;3 isoforms.

## Material and methods

### Plant material and growth conditions

The *gln1;1*, *gln1;2*, *gln1;3*, *gln1;4*, and *gln1;5* T-DNA mutants of *Arabidopsis thaliana* used in this study are listed in Supplementary Table S1 at *JXB* online. Double- and triple-mutant lines were obtained by crossing single-mutants, and genotyping was performed on the third generation. Single-mutants were backcrossed to the N60000 wild-type Col-0 accession. Homozygous lines were selected from genotyping through PCR on the genomic DNA (primers are listed in Supplementary Table S2). For each *GLN1* gene, two or three different mutant alleles were obtained. The absence of mRNA was verified using RT-PCR and RT-qPCR to confirm knock-out mutants (see Supplementary Fig. S1). The *gln1;1-gln1;2* double-mutant was obtained from the cross of GK-265C09 and SALK_102291, *gln1;1-gln1;3* from the cross of GK-265C09 and SALK_072283, and *gln1;2-gln1;3* from the cross of SALK_102291 and SALK_072283. The *gln1;1-gln1;2-gln1;3* triple-mutant was obtained from the cross of *gln1;1-gln1;3* and *gln1;1-gln1;2*. Note that the *gln1;2* SALK_102291 mutant had been previously characterized by our group (Lothier *et al.*, 2011).

#### Phenotyping and gene expression studies

Seeds were systematically stratified for 48 h in 0.1% agar solution (in water) in the dark at 4°C before sowing, ensuring that homogeneous germination occurred 2 d after sowing (DAS). Plants were grown in soil in pots under control conditions in growth chambers with a photon flux density of 160 µmol m^–2^ s^–1^ under short-day (8 h light) or long-day (16 h light) conditions. Pots were watered with solutions containing 2 mM or 10 mM nitrate according to Lemaître *et al.*, (2008).

#### ^15^N-tracing experiments

Seeds were sown on sand to perform tracing experiments as described previously by Masclaux-Daubresse and Chardon (2011). Sowing was performed in order to obtain either one plant per pot (in a central position) or six plants positioned in a circle, as described by Diaz *et al.* (2006). Plants were grown under short-day conditions (8 h light; 160 µmol m^–2^ s^–1^) and transferred to long-days at 56 DAS (16 h light; 160 µmol m^–2^ s^–1^), as described by Masclaux-Daubresse and Chardon (2011).

### RNA isolation, reverse transcription, and qPCR analysis

Total RNA was isolated using TRIzol® reagent (Life Technologies, Saint-Aubin, France) and reverse transcription was performed using M-MuLV reverse transcriptase (ThermoFisher Scientific, Villebon-sur-Yvette, France) and oligo(dT) (Eurofins MWG Operon, Ebersberg, Germany), after DNaseI treatment (ThermoFisher Scientific). Total RNA was used for both semi-quantitative RT-PCR and for real-time RT-PCR using a CFX thermocycler (Biorad, Marnes-La-Coquette, France) and Mesa FAST qPCR Mastermix Plus for the SYBR assay (Eurogentec, Angers, France). The following were used as reference genes (all of which showed stable expression): adenosine phosphoribosyl transferase (*APT*, At1g27450), ELONGATION FACTOR 1 (*EF1α4*, At5g60390), and actin (At3g18780). Gene-specific primer couples are listed in Supplementary Table S3.

### Construction of *ProGLN1::uidA* and *ProGLN1::GFP* plants, and microscopy

Amplification of approximately 2000 bp of each of the *GLN1* promoters was performed using Phusion high-fidelity DNA polymerase (ThermoFisher Scientific) and the sequences of the cloned promoters were verified. Primers are listed in Supplementary Table S3. PCR products were cloned in the pGWB3 and pGWB4 vectors (Nakagawa *et al.*, 2007) using Gateway technology (Invitrogen, Carlsbad, CA, USA). Transformants in the Col background were obtained by floral dipping (Clough and Bent, 1998) and selected for their kanamycin resistance. More than 12 transformants were obtained for each construct and tested for β-glucuronidase (GUS) staining and expression of green fluorescent protein (GFP). Only three selected representative lines were used in all our experiments.

Plants carrying the *ProGLN1::uidA* construct were stained for 24 h in 50 mM KH_2_PO_4_, pH7, 0.05% (v/v) Triton X-100, 5 mM ferricyanide, 5 mM ferrocyanide, and 5-bromo-4-chloro-3-indolyl-ß-D-glucuronic acid (X-GlcA) at 1 mg ml^–1^. The plants were then destained in ethanol. Entire plants were observed under a magnifying glass, and leaf sections (8 µm thick) under a light microscope (DMRB, Leica Microsystems) after cutting using a Zeiss microtome.

Leaves of plants carrying the *ProGLN1::GFP* construct were excised and clear images of the vein system were obtained by using a razor blade to remove the overlying cell layers (corresponding to the epidermis and spongy mesophyll) from the abaxial side of the leaf, as described by Cayla *et al.* (2015). Observations were carried out within a few minutes of preparation, by confocal laser-scanning microscopy (SP2 Leica) and with an Axiozoom V16 (Zeiss) microscope equipped with a Plan-Neofluar Z 2.3x/0.57 RWD 10.6 mm.

For *in situ* hybridization, plants were fixed in 4% formaldehyde (fresh from paraformaldehyde) in PBS under vacuum for 3 × 20 min, and left in fixative overnight. After fixation, the plants were washed, dehydrated, and embedded in paraffin, essentially as described by Jackson (1991). Paraffin sections (8 µm thick) were cut with a disposable metal knife and attached to precoated glass slides (Fisher Scientific, USA). Specific sense and antisense probes of *GLN1* genes were synthesized using the Riboprobe^®^ combination system T3/T7 (Promega, Charbonnières, France) and digoxigenin (DIG-UTP; Roche Diagnostics, Meylan, France) on PCR products (see Supplementary Table S3) according to the manufacturer’s instructions. Probe specificities were verified by performing dot blots with three dilutions (1/10, 1/100, and 1/1000) of each of the different *GLN1* PCR products. *In situ* hybridization was carried out as described by Nikovics *et al.*, (2006) and the hybridization temperature was 43 °C. Immunodetection of the DIG-labelled probes was performed using an anti-DIG antibody with alkaline phosphatase as instructed by the manufacturer (Roche Diagnostics, Meylan, France). The coloration reaction was done using BCIP/NBT Color Development Substrate (Promega, Charbonnières, France). Observations were performed using an Axiozoom V16 (Zeiss) microscope equipped with a Plan-Neofluar Z 2.3x/0.57 RWD 10.6 mm.

### Determination of total N and C contents, and ^15^N abundance and partitioning in the tracing experiment

A pulse-chase experiment using ^15^N was performed in order to assess the N remobilization from rosette to seeds in the different mutant lines. Plants were grown and labelled at the vegetative stage similar to as described by Masclaux-Daubresse and Chardon (2011). Briefly, labelled nutrient solution was supplied for 24 h at 42 DAS. After labelling, and until the end of the life cycle, plants were watered with non-labelled nutritive solution. Harvesting of the different components (rosette, seeds, and stems; the latter including the stem, silique envelopes, and cauline leaves) was performed at seed maturity. All the plant material was dried, ground, and subsamples of 1000–2000 mg were carefully weighed in tin capsules. N and C contents and ^15^N abundance were determined using a FLASH 2000 Organic Elemental Analyzer (ThermoFisher Scientific) coupled to a DELTA V ADVANTAGE isotope ratio mass spectrometer (ThermoFisher Scientific). The ^15^N abundance was defined as A% = 100(^15^N)/(^15^N+^14^N) for labelled plant samples and for unlabelled control plants (A%_control_ was 0.36052). The ^15^N enrichment was calculated as (A%_sample_ – A%_control_). The absolute quantity of ^15^N contained in a sample *i* was *Q*_*i*_ = DW_*i*_ × (A%_*i*_ – A%_control_) × %N_*i*_, where %N_*i*_ = 1/[mg N × (100 mg DW_*i*_)]. The *Q* value of the whole plant (wp) was calculated as *Q*_wp_ = ∑*Q*_*i*_. The partitioned ^15^N in an organ *i* was then calculated as the ^15^N harvest index, ^15^NHI = [(A%_*i*_ – A%_control_) × DW_*i*_ × %N_*i*_]/[(A%_wp_ – A%_control_) × DW_wp_ × %N_wp_] = *Q*_*i*_/*Q*_wp_.

### Glutamine synthetase activity and western blots

Enzymes were extracted from frozen leaf material (150 mg FW) that had been stored at –80°C. GS activity was determined using the γ-glutamyl transferase method, and GS western blots for the GS1 and GS2 proteins were prepared according to Lothier *et al.* (2011). Antibodies raised against the GS1 and GS2 isoenzymes were produced by Lemaître *et al.* (2008). Quantification of signals on the western blot membranes was performed using the Multi Gauge 3.2 software (Fujifilm).

The relative proportions of GS activity due to the chloroplastic and cytosolic isoforms were evaluated after fractionation. Plant material was extracted (2 g FW in 10 ml extraction buffer) according to Lothier *et al.* (2011). After centrifugation (15 000 *g* 15 min at 4°C), the supernatant was filtered (0.2-µm filter, GelmanSciences) and injected into a Mono Q anion exchange column (5/50 GL, GE Healthcare) attached to a FPLC system (ÄKTApurifier, GE Healthcare). The Mono Q column had been pre-equilibrated with 30 ml of equilibration buffer (25 mM Tris-HCl, 1 mM MgCl_2_, 1 mM EDTA) before loading. FPLC was performed at room temperature. Protein fractions were eluted from the column using a linear gradient from 0.1 to 0.7 M NaCl with a flow rate of 1.0 ml min^–1^. Fifty fractions (500 µl) were collected and assayed for GS activity. Aliquots were denatured for SDS-PAGE and western blotting assays.

### Phloem sap exudation

Phloem exudates were collected from the petioles of mature leaves harvested at midday using a method adapted from Tetyuk *et al.* (2013) and King and Zeevaart (1974). The exudate of the sixth leaf of each plant was collected over a period of 2 h. Petioles were sectioned, recut in a Petri dish filled with exudation buffer (10 mM HEPES, 10 mM EDTA, pH 7.5) and then immersed in 80 µl of the same buffer in a 0.5-ml Eppendorf tube. To avoid contamination from the surrounding tissues, the sap was allowed to exude for 2 minutes in the cutting dish before quickly being transferred to the Eppendorf tube. Twelve biological replicates were used for each genotype and each nutrition treatment. Amino acid measurements were performed on pooled samples of two exudates. The fresh weight of each leaf used for exudation was measured and the exudates were stored at –80 °C prior to use.

### Measurement of amino acids and ammonium

Free amino acids and ammonium were extracted by grinding frozen material in 2% 5-sulfosalicylic acid (100 mg FW ml^–1^). After centrifugation (13 000 *g*), the supernatant was collected in fresh tubes and buffered to pH 2.2 with saturated LiOH before chromatography. Free amino acids and the ammonium trapped by the low-pH buffer were determined using an Amino Tac JLC-500/V amino acid analyser (JEOL, Croissy sur Seine, France) according to manufacturer’s instructions and the software provided. Individual amino acids were summed to give the total content.

### Accession numbers

Sequence data can be found in the GenBank/EMBL libraries under the following accession numbers: At1g66200, At1g48470, At3g17820, At5g16570, At5g37600.

## Results

### The *gln1;1-gln1;2-gln1;3* triple-mutation is required to significantly affect N remobilization to the seeds

The different single, double, and triple homozygous mutants (combining *gln1;1*, *gln1;2*, and *gln1;3*) isolated in the Col background (Supplementary Fig. S1) were labelled with ^15^N at the vegetative stage. ^15^N-tracing was performed as previously described by Masclaux-Daubresse and Chardon (2011), and as previously done by Lothier *et al.* (2011) on a *gln1;2* mutant in the *Wassilewskija* (WS) background. Labelling was performed at an early stage of plant development (42 DAS under short days), well before flowering, in order to ensure that all the ^15^NO_3_^–^ taken up from the soil was assimilated by the plants into organic N-containing molecules. The ^15^N solution was kept in contact with the plant roots for 24 h. Four plants per genotype were harvested 24 h after the ^15^N-labelling period to confirm that uptake was not significantly different between plant repeats or between genotypes (data not shown).

The remaining plants were harvested at the end of their life cycle to measure total seed yield, biomass, N content, and ^15^N enrichment (Masclaux-Daubresse and Chardon, 2011; Guiboileau *et al.*, 2012). The data obtained were then used to calculate the harvest index (HI; g of seeds g^–1^ of total dry matter), the nitrogen harvest index (NHI; g of N g^–1^ of total dry matter), which corresponds to the quantity of N in the seeds compared to the total N within the plant, and the ^15^N harvest index (^15^NHI; g of ^15^N g^–1^ of total dry matter), which corresponds to the quantity of ^15^N in the seeds compared to the total ^15^N within the plant. NHI and ^15^NHI are indicators of the nitrogen utilization and N remobilization efficiencies, respectively (Chardon *et al.*, 2012; Havé *et al.*, 2017). The ^15^N-tracing experiments performed on *gln1* single- and double-mutants showed that there were no significant differences relative to the wild-type for HI, NHI, or ^15^NHI (Supplementary Figs S2, S3), and thus no N-remobilization defects in any of the GS single- or double-mutants (note that although only the *gln1;2* Col mutant is presented here, similar results were obtained with the *gln1;2* WS mutant by Lothier *et al.*, 2011).

**Fig. 1.**
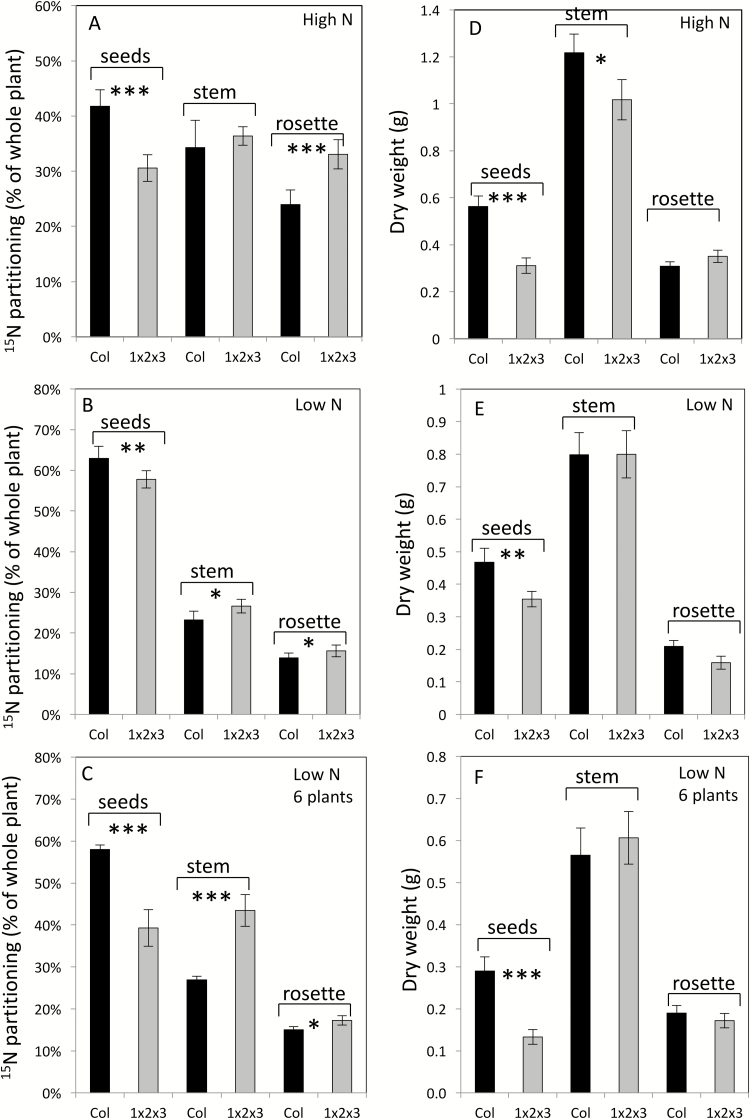
N-remobilization efficiency and seed yield are reduced in the Arabidopsis *gln1;1-gln1;2-gln1;3* triple-mutant. ^15^N partitioning in the seeds, stems, and rosettes of the wild-type (Col; black bars) and the triple-mutant (1x2x3; grey bars) were calculated at harvest from measurements of dry weight, N contents, and ^15^N abundances. The wild-type and mutant were initially grown in sand under short-day and ^14^N conditions, then pulse-labeled at 42 d after sowing (DAS) with ^15^NO_3_^–^ for 24 h and then further grown under ^14^N conditions until harvest. At 56 DAS, plants were transferred to long-days for flowering. Plants were grown under high-nitrate conditions (A, D) or under low-nitrate conditions (B, C, E, F). In (C, F) the plant density was increased from one plant per pot to six in order to increase nitrate limitation. Data are means (±SE), *n*=6–8 plants. Significant differences between the mutant and the wild-type as determined by Student’s *t*-test are indicated: *P<0.05, **P<0.01, ***P<0.001. Experiments were repeated 2–3 times.

In contrast, the *gln1;1-gln1;2-gln1;3* triple-mutant (referred to as 1x2x3 in Fig. 1) displayed significantly lower ^15^NHI partitioning to the seeds relative to the wild-type under both high- (Fig. 1A) and low- (Fig. 1B, C) nitrate conditions (see also Supplementary Fig. S3). The decrease of ^15^NHI in the triple-mutant was clearly exacerbated when plant density was increased (from one to six plants per pot) under low-nitrate conditions (Fig. 1C). The decrease in the triple-mutant was due to the retention of ^15^N in the vegetative tissues, either in the rosette (Fig. 1A–C) and/or in the stems (Fig. 1B, C).

**Fig. 2.**
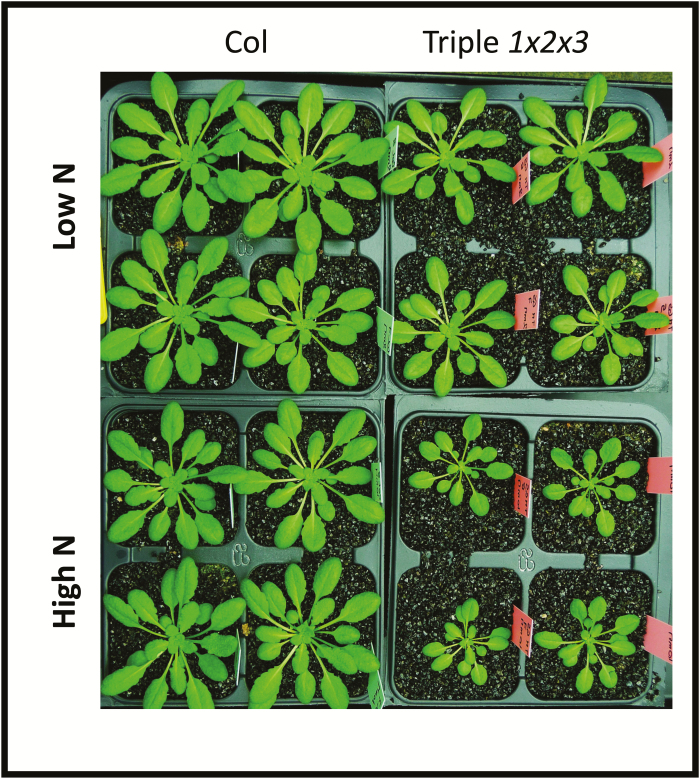
The *gln1;1-gln1;2-gln1;3* triple-mutant is smaller under both low- and high-nitrate conditions. The mutant (Triple 1x2x3) and wildtype (Col) were grown under low- and high-nitrate conditions for 35 d in short-day conditions.

As a result of their lower efficiency of N translocation to the seeds, the total seed yield (DW of seeds) of the triple-mutant was significantly lower than in the wild-type (Fig. 1D–F, Supplementary Fig. S3). Lower total seed yield was observed under both low- and high-nitrate conditions. Individual seeds of the triple-mutant were significantly lighter compared to the wild-type (*P*<0.01, Student’s *t*-test, *n*=6 individual plants): the dry weight of one seed in the mutant was 13.4 ± 0.8 µg and 12.5 ± 0.4 µg under low- and high-nitrate conditions, respectively, compared with 17.2 ± 0.9 µg and 14.9 ± 1.4 µg in the wild-type, i.e. the difference was greater under nitrate limitation. However, no change in N concentration was detected between the wild-type and the triple-mutant, and the C/N ratio was maintained. The triple-mutant plants had smaller rosettes than wild-type under both high- and low-nitrate (Fig. 2, Supplementary Fig. S4).

### The leaves of the *gln1;1-gln1;2-gln1;3* triple-mutant contain greater total amino acid and ammonium contents than the wild-type

**Fig. 3.**
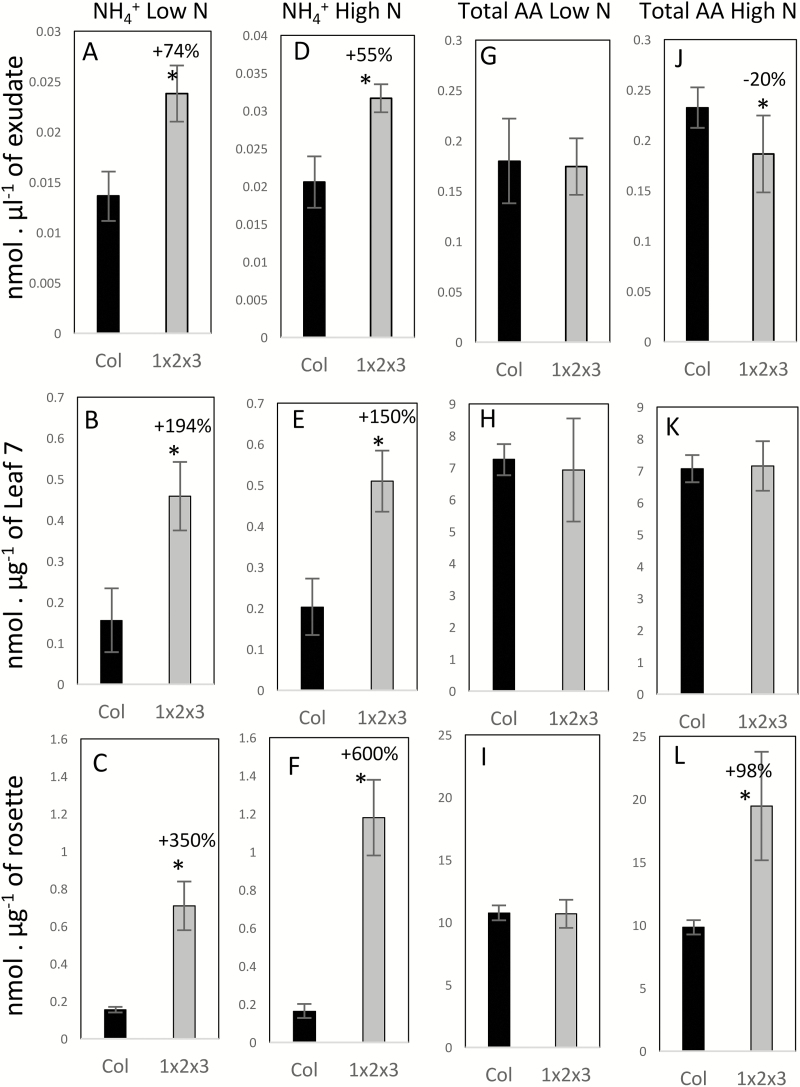
Ammonium and amino acid concentrations are affected by the *gln1;1-gln1;2-gln1;3* triple-mutation. Ammonium concentrations (A–F) and total free amino acid concentrations (G–L) were determined in the phloem exudates (A, D, G, J), in the 7th leaf (B, E, H, K), and in the rosettes (C, F, I, L) of the wild-type (Col, black bars) and the triple-mutant (1x2x3, grey bars) plants grown under low (A–C, G–I) or high (D–F, J–L) nitrate conditions for 45 d. Data are means (±SD), *n*=5–6 plants. Significant differences between the mutant and the wild-type as determined by Student’s *t*-test are indicated: *P<0.05. The percentage increase or decrease is indicated when significant.

At 45 DAS, the 6th and 7th leaves of the rosettes are sources for nitrogen (Diaz *et al.*, 2008; Avila-Ospina *et al.*, 2014), and consequently phloem exudates were collected from these leaves at this time. The rest of the rosette was also harvested. The phloem exudates and the rosettes of the triple-mutant contained higher ammonium concentrations than the wild-type in both low- and high-nitrate conditions (Fig. 3A–F). Since there was no significant difference in the fresh weight of the 6th leaf between the triple-mutant and the wild-type (P>0.1, *t*-test, *n*=12), the concentrations of the amino acids and ammonium in the phloem exudates are reported simply as nmol µl^–1^. Total amino acid concentrations were generally not significantly different between the mutants and the wild-type (Fig. 3G–I, K), except in the rosettes (Fig. 3L) and in the phloem exudates under high-nitrate conditions (Fig. 3J). When grown under high-nitrate conditions, the triple-mutant accumulated a greater concentration of amino acids in the rosettes and released less amino acids in the phloem exudates compared to the wild-type, thus revealing the presence of defects in amino acid export.

**Fig. 4.**
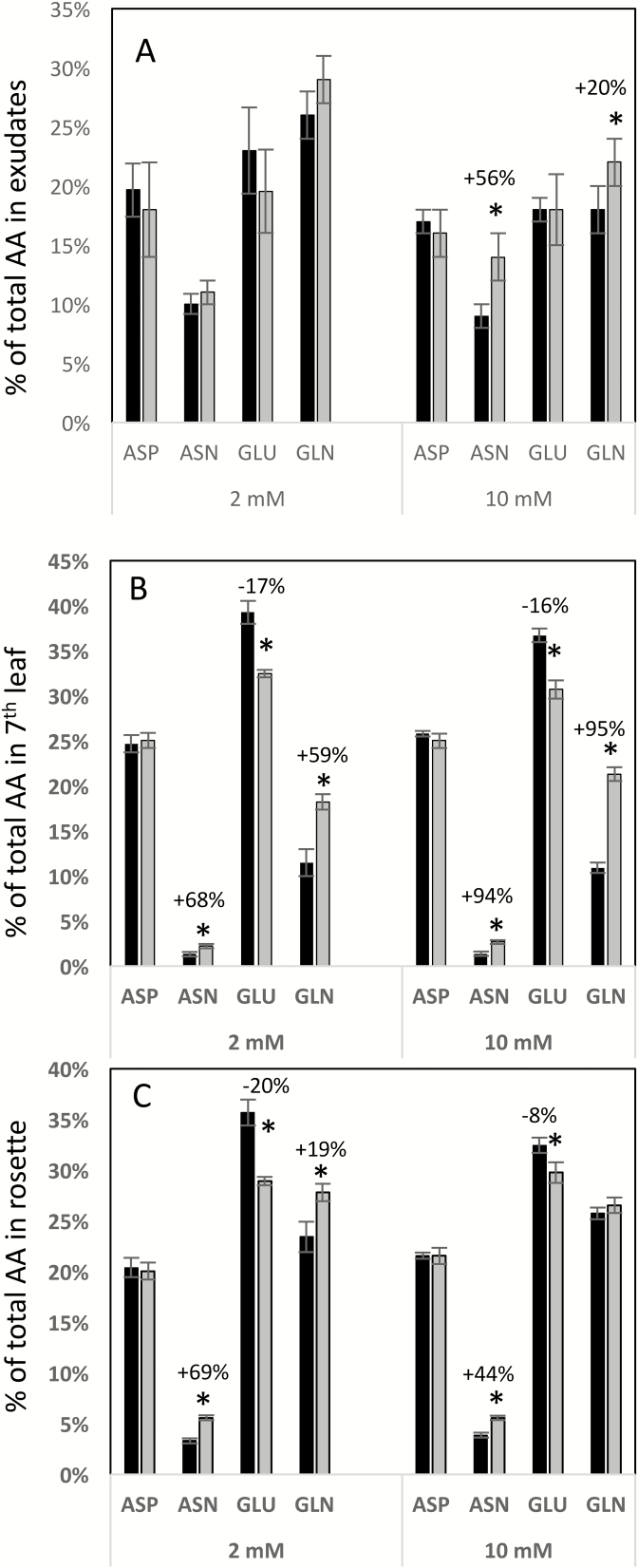
Individual amino acid proportions are different in the *gln1;1-gln1;2-gln1;3* triple-mutant. Individual amino acid proportions (as percentage of total free amino acids) were determined in the phloem exudates (A), the 7th leaf (B), and the rosette (C) of wild-type (black bars) and the triple-mutant (grey bars) plants grown under low (2 mM) or high (10 mM) nitrate conditions for 45 d. Data are means (±SD), *n*=5–6 plants. Significant differences between the mutant and the wild-type as determined by Student’s *t*-test are indicated: *P<0.05. The percentage increase or decrease is indicated when significant.

Determination of the composition of individual amino acids revealed that the percentage of asparagine was strongly increased in the rosette and the 7th leaf of the triple-mutant compared with the wild-type, under both low- and high-nitrate conditions (Fig. 4B, C, Supplementary Table S4). The percentage of glutamine was also increased in the 7th leaf of the triple-mutant under both low and high nitrate, but the increase in glutamine in the rosette of the triple-mutant was much more modest than that of asparagine, and only significant under low nitrate (Fig. 4B, C). Accordingly, the percentage of glutamate was decreased in both the rosette and the 7th leaf of the mutant.

Although the compositions of individual amino acids in the leaf tissues of the triple-mutant were generally different to the wild-type under both nitrate treatments (Fig. 4B, C), in the phloem exudates only the percentages of asparagine and glutamine under high-nitrate conditions were different (Fig. 4A), with the mutant having higher contents of both compared with the wild-type.

### The *in vitro* total activity of glutamine synthetase and the GS1 protein content are sharply decreased in the *gln1;1-gln1;2-gln1;3* triple-mutant

**Fig. 5.**
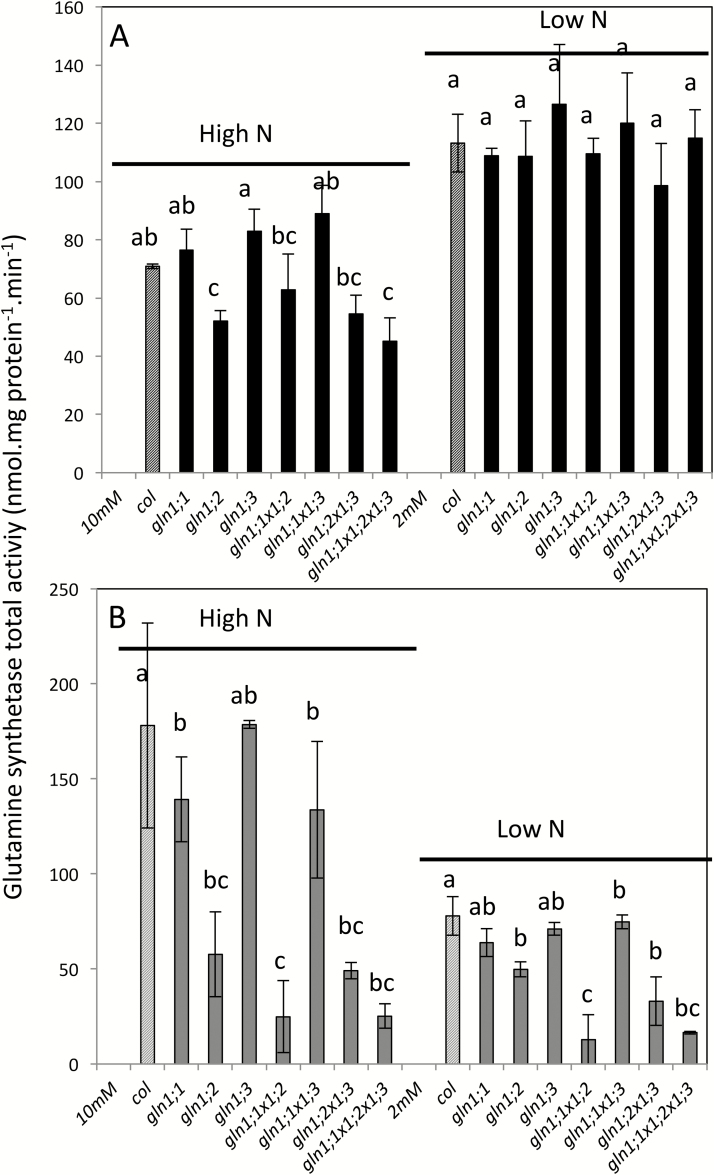
Total glutamine synthetase activity in GS1 mutants. The wild type (Col, dashed bars) and mutants (solid bars) were grown under short-day conditions for 35 d with high (10 mM) or low (2 mM) nitrate. Rosettes (A) and roots (B) were harvested separately for measurement of GS transferase activity. Data are means (±SD), *n*=4 plants. Different letters indicate significant differences between genotypes (*P*<0.05) as determined using XLSTAT ANOVA Newman–Keuls (SNK) comparisons. Comparisons were done separately for low N and high N.

The GS activity measured *in vitro* was the sum of the GS1 and GS2 activities. In the wild-type and in all the GS1 mutants, the GS activity was higher under low-nitrate conditions than under high-nitrate in the shoot, and in contrast was higher under high-nitrate conditions relative to low-nitrate in the roots (Fig. 5). GS activity was not significantly different in the single, double, or triple *gln1* mutants compared to the wild-type, except for those carrying the *gln1;2* mutation. Under high-nitrate conditions, GS activity was significantly decreased in both roots and shoots of the *gln1;2* single, double, and triple mutants (i.e. *gln1;2*, *gln1;1-gln1;2*, *gln1;2-gln1;3*, and *gln1;1-gln1;2-gln1;3*). Under low-nitrate nutrition, GS activity was decreased only in the roots of the *gln1;2* single, double, and triple mutants, while it was not different in the shoots.

The GS1 protein contents were strongly affected in all the *gln1;2* single, double, and triple mutants, both in shoots and roots and under low- and high-nitrate conditions (Supplementary Fig. S5). The reason why the total GS activity was unchanged in the shoots of the *gln1;2* mutants under low-nitrate while the GS1 protein content showed a decrease can be explained by the fact that GLN1;2 has low affinity for ammonium (Ishiyama *et al.*, 2004). Nevertheless, the lower GS1 activity in roots under both low- and high-nitrate conditions could explain the smaller rosette size observed in the triple-mutant, (Konishi *et al.*, 2017, 2018).

### Chloroplastic glutamine synthetase (GS2) and asparagine synthetase 2 (ASN2) are induced in the *gln1;1-gln1;2-gln1;3* triple-mutant

In order to determine why the relative proportions of glutamine and asparagine were higher in the triple-mutant than the wild-type (Fig. 4), we first examined the expression levels of the remaining intact GS1 genes (*GLN1;4* and *GLN1;5*), the chloroplastic GS2 gene (*GLN2*), and the three asparagine synthetase genes (*ASN1*, *ASN2*, *ASN3*).

**Fig. 6.**
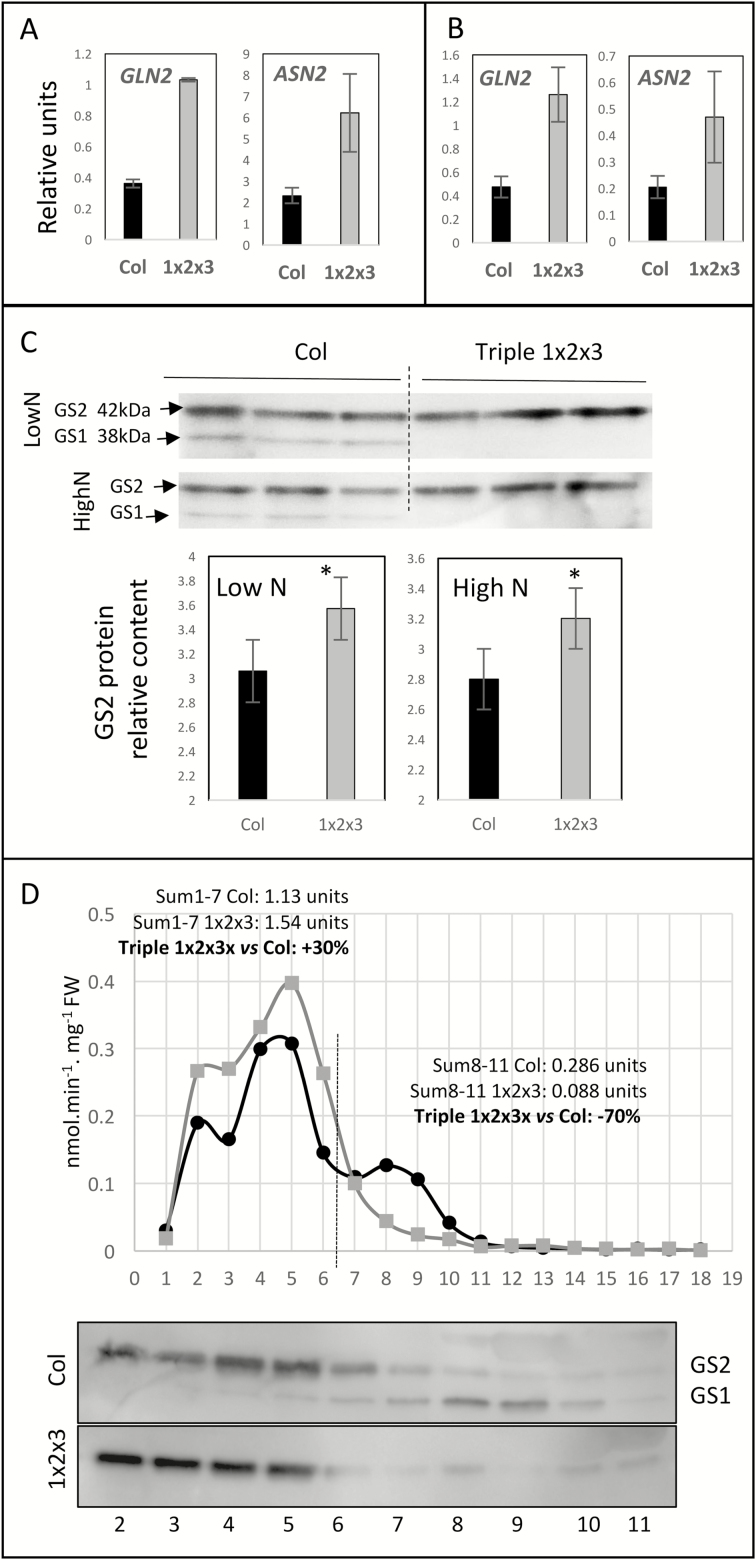
The expression levels of *ASN2* and GS2 are increased in the *gln1;1-gln1;2-gln1;3* triple-mutant. (A, B) *ASN2* and *GLN2* expression levels were measured through RT-qPCR in the 7th leaf (A) and the rosettes (B) of wild-type (Col, black bars) and the triple-mutant (1x2x3, grey bars) plants grown under low (A) or high (B) nitrate conditions for 45 d. Gene expression levels were normalized to a synthetic reference gene that combines the Actin and APT *C*_t_ values as √(*C*_t,Actin_×*C*_t,APT_). Data are means (±SD), *n*=3 plants, with three technical repeats each. Significant differences between the mutant and the wild-type as determined by Student’s *t*-test are indicated: *P<0.05. (C) Western blots were performed using GS antibodies on rosettes of wild-type (Col, black bars) and the triple-mutant (1x2x3, grey bars) plants grown under low N or high N conditions. Both the GS1 and GS2 isoforms were detected in crude extracts. Equal amount of proteins (5 µg) were loaded in each lane. The histograms present the quantification of GS2 signal intensity. Data are means (±SD), *n*=3 plants. (D) The GS2 and GS1 isoforms were separated by anion exchange chromatography. GS activities were measured in the eluted fractions from crude extracts for the wild-type (Col, black circles) and the triple-mutant (1x2x3, grey squares). The GS1 and GS2 contents were detected using western blots after SDS-PAGE separation. GS1 was mainly present in fractions 6–10. Equal volumes were loaded in each lane.

There were no differences in the expression levels of *GLN1;4*, *GLN1;5*, *ASN1*, and *ASN3* between the wild-type and the triple mutant under either low- or high-nitrate conditions (data not shown). By contrast, the expression levels of *GLN2* and *ASN2* were significantly increased relative to the wild-type in the rosettes of the triple-mutant under high nitrate (Fig. 6A), and in the 7th leaf of the triple-mutant under low nitrate (Fig. 6B).

Quantification of protein levels determined on western blots using GS antibodies showed that the GS2 protein content was significantly higher in the triple-mutant than in the wild-type, under both low and high nitrate (Fig. 6C), confirming the tendency that was observed in Supplementary Fig. S5. In addition, separation of the GS2 and GS1 isoforms by ion-exchange chromatography showed that the cumulated value of the GS activities measured in the GS2-containing fractions (fractions 1–6) was higher in the triple-mutant than in the wild-type (+30%), (Fig. 6D).

### The different *GLN1* genes are differentially expressed depending on the plant organ, the photoperiod, and the nitrate conditions

The induction of *GLN1;1*, *GLN1;*2, *GLN1;3*, and *GLN1;4* during leaf senescence has previously been reported in several studies (Bernhard and Matile, 1994; Diaz *et al.*, 2008; Lothier *et al.*, 2011; Avila-Ospina *et al.*, 2014). Here, the quantitative real-time RT-qPCR conducted on different organs (whole rosette, stem, flowers) of the Col wild-type showed that the five *GLN1* genes were differentially expressed during rosette ageing, depending on nitrate availability and on the photoperiod (Supplementary Fig. S6). In all the conditions, the *GLN1;1* and *GLN1;2* transcript levels were high in the rosette and stem tissues (Supplementary Fig. S6A, B) while the *GLN1;5* expression level was weak, except in the flowers (Supplementary Fig. S6E). *GLN1;3* was also expressed at a higher level in the flowers than in the rosette and stem (Supplementary Fig. S6C). Consistent with their patterns of senescence, the expression levels of *GLN1;1*, *GLN1;2*, and *GLN1;4* were increased with ageing in the rosettes, but this was only significant under short-day conditions (Supplementary Fig. S6A, B, D). The expression levels were also higher in older stem tissues relative to younger ones under long-day conditions, i.e. at the bottom of the stem compared to the top. *GLN1;1* and *GLN1;4* were preferentially expressed under low-nitrogen conditions. *GLN1;1* and *GLN1;4* were also preferentially expressed in the roots under low-nitrogen conditions (Supplementary Fig. S6F).

### The five *GLN1* isoforms are all expressed in companion cells but not in veins of the same order

**Fig. 7.**
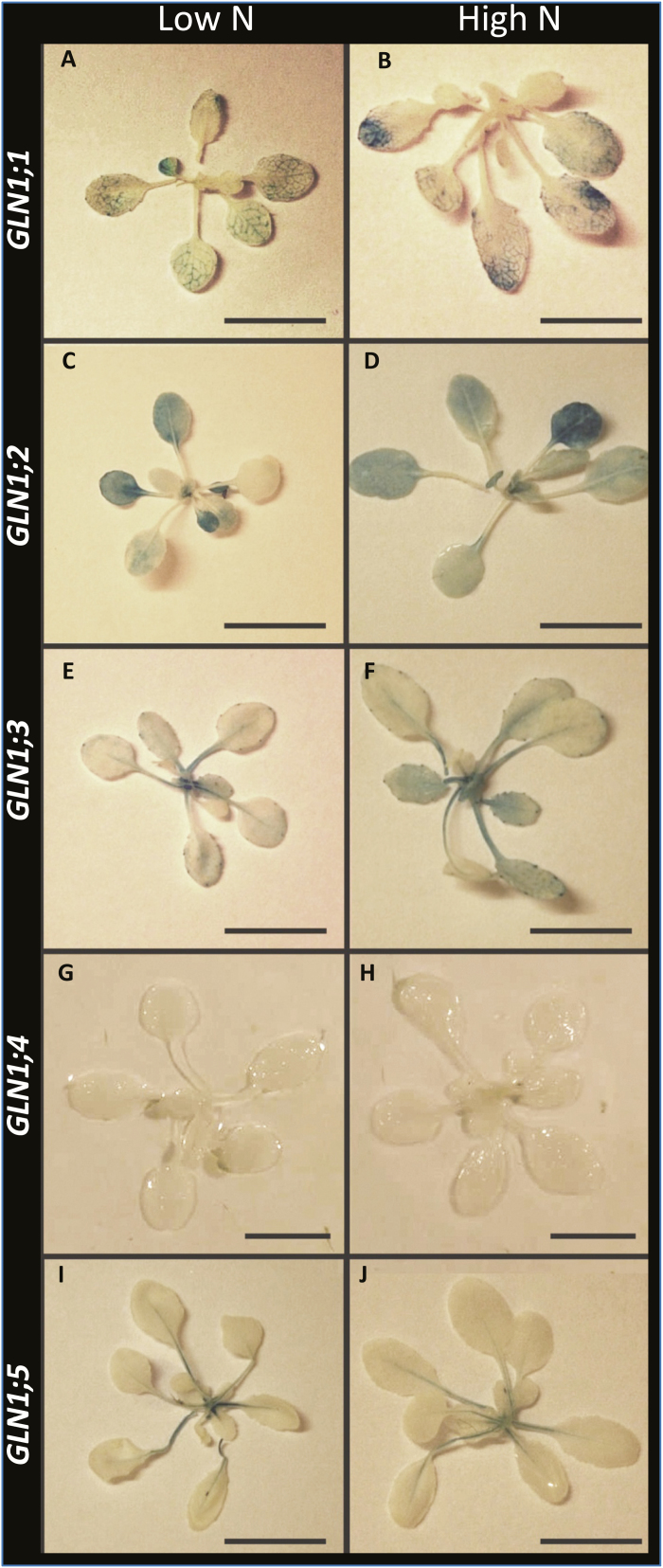
Patterns of *GUS* expression driven by the promoters of the *GLN1* genes in young plants. Transgenic lines were transformed with transcriptional fusions between the *GLN1* promoters and the *uidA* reporter gene. GUS staining was observed in the rosettes of 20-d-old plants grown under long days and either low- or high-nitrate conditions. (A, B) *ProGLN1;1:uidA*, (C, D) *ProGLN1;2:uidA*, (E, F) *ProGLN1;3:uidA*, (G, H) *ProGLN1;4:uidA*, and (I, J) *ProGLN1;5:uidA*. Scale bars are 1 cm.

Plants carrying *GLN1* promoters fused to the *uidA* reporter gene (*ProGLN1::uidA*) were used to localize the different promoter activities. All the promoter activities were detected in the same tissues under low- and high-nitrate conditions (Fig. 7). GUS staining was easily detected in all the *ProGLN1::uidA* transformants, except in the *ProGLN1;4::uidA* plants, despite the numerous lines of this type that were obtained. The functionality of the *ProGLN1;4::uidA* construct was validated, as a blue signal was observed in the stamen of transformants (Supplementary Fig. S7).

**Fig. 8.**
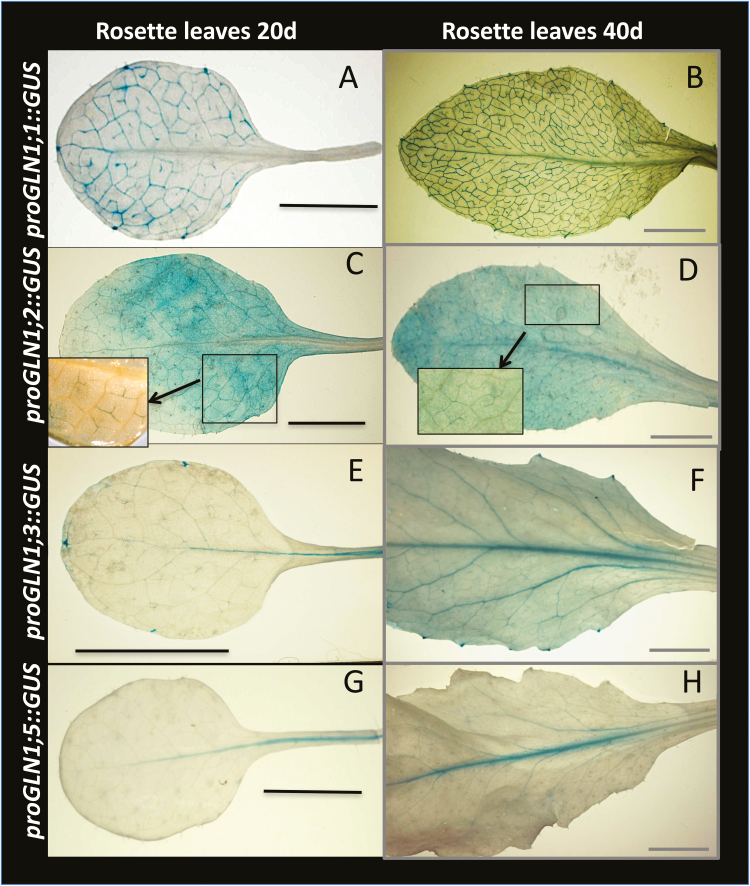
Patterns of *GUS* expression driven by the promoters of the *GLN1* genes in young and old leaves. The images show GUS staining in rosette leaves of 20-d-old and 40-d-old plants grown under high nitrogen and long days for *ProGLN1;1:uidA* (A, B), *ProGLN1;2:uidA* (C, D), *ProGLN1;3:uidA* (E, F), and *ProGLN1;5:uidA* (G, H). In all cases the blue signal is located in the veins. Similar localization was observed in plants grown with low N (not shown). No signal could be observed for *ProGLN1;4:uidA.* Scale bars are 1 cm.

While all the *ProGLN1::uidA* fusions (except *ProGLN1;4::uidA*)demonstrated GUS activity in the leaf vasculature (Fig. 8), it was interesting to note that the blue signal was localized in different-order veins depending on the promoters. We classified the different veins by their hierarchical order according to Sack and Scoffoni (2013), dividing them into low-order (main) veins, i.e. the mid-vein (or first-order) and secondary (second-order) veins, and high-order veins, i.e. the minor veins (third-, fourth-, fifth-order). *ProGLN1;1::uidA* activity was mainly detected in the second- and third-order veins whilst *ProGLN1;2::uidA* activity was located in mesophyll cells and in the high-order veins (third- and fourth-order). *ProGLN1;3::uidA* activity was mainly located in the first-order vein (mid-vein) and it appeared in the second-order veins of older leaves. Although weakly expressed in the leaves, *ProGLN1;5::uidA* activity was detected in the first-order veins. Using microtome transversal sections and light microscopy (Supplementary Fig. S8), we confirmed that all the *ProGLN1::uidA* activities were present in the vascular tissues, and more precisely at the phloem poles. Microscopy also allowed us to detect a weak *ProGLN1;4::uidA* blue signal in the phloem of the high-order veins.

**Fig. 9.**
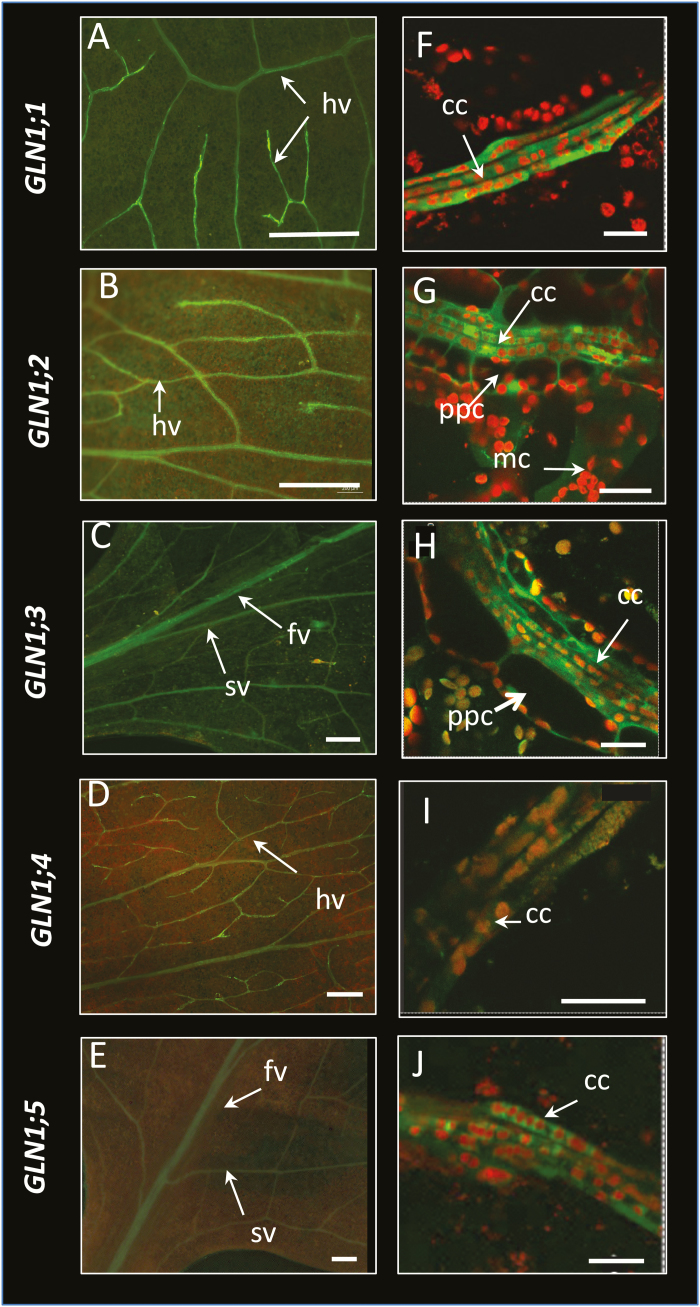
Fluorescence zoom microscope images and confocal laser-scanning images of GFP expression patterns in leaves. Zoom microscope images (A–E) show that *ProGLN1;1::GFP*, *ProGLN1;2::GFP*, and *ProGLN1;4::GFP* were mainly expressed in high-order veins (hv) while *ProGLN1;3::GFP* and *ProGLN1;5::GFP* were expressed in the first-order veins (fv) and second-order veins (sv). A strong GFP background was detected in the leaf blade in the *ProGLN1;2::GFP* plants. Scale bars are 0.5 mm. Confocal laser-scanning images (F-J) show that *ProGLN1;1::GFP*, *ProGLN1;4::GFP*, and *ProGLN1;5::GFP* were exclusively expressed in the companion cells (cc) of veins. *ProGLN1;2::GFP* was expressed in the companion cells, phloem parenchyma cells (ppc), and mesophyll cells (mc), and *ProGLN1;3::GFP* was expressed in companion cells and phloem parenchyma cells. Observations were performed with Leica SP5 Tandem confocal microscope. Scale bars are 20 µm.

The fluorescence of the *ProGLN1::GFP* constructs observed in fresh tissues under an Axiozoom microscope confirmed that the *GLN1;1*, *GLN1;2*, and *GLN1;4* promoters were active in high-order veins (Fig. 9A, B, D) and that the *GLN1;3* and *GLN1;5* promoters were active in the first-order veins, and additionally in the second-order veins for *GLN1;3* (Fig. 9C, E). For confocal laser-scanning microscopy, the epidermis was removed from the leaves to image the vascular tissues (Cayla *et al.*, 2015). *ProGLN1::GFP* fusions were expressed in the companion cells (Fig. 9F–J), which were identified based on their typical and characteristic chloroplast alignments (Cayla *et al.*, 2015). The *ProGLN1;1::GFP*, *ProGLN1;4::GFP*, and *ProGLN1;5::GFP* fusions were exclusively localized in the companion cells (Fig. 9F, I, J). *ProGLN1;3::GFP* was also in the phloem parenchyma cells surrounding the companion cells (Fig. 9H) and *ProGLN1;2::GFP* was in the phloem parenchyma and the mesophyll cells (Fig. 9G) in addition to companion cells. The qRT-PCR results obtained from the stems and flowers were then confirmed *in vivo* using the different *ProGLN1::uidA* and *ProGLN1::GFP* fusions (Supplementary Figs S7, S9, respectively). *In situ* hybridization of the *GLN1* mRNA with specific antisense probes confirmed the localization of *GLN1;1*, *GLN1;2*, and *GLN1;4* in the phloem poles of the high-order veins, and the localization of *GLN1;3* and *GLN1;5* in the phloem poles of the first-order veins (Supplementary Fig. S10).

## Discussion

Studies of the *GS1* gene family in barley (*Hordeum vulgare*), maize (*Zea mays*), wheat (*Triticum aestivum*), rice (*Oriza sativa*), oilseed rape (*Brassica napus*), and Arabidopsis have shown that the GS1 isoforms are differentially expressed depending on the organ, the developmental stage, and the nitrogen availability, and that they play different roles in determining seed size and number, and in plant growth capacity (Martin *et al.*, 2006; Bernard *et al.*, 2008; Cañas *et al.*, 2010; Lothier *et al.*, 2011; Funayama *et al.*, 2013; Guan *et al.*, 2015; Xie *et al.*, 2015; Guan and Schjoerring, 2016; Konishi *et al.*, 2017). *GLN1* genes from monocots and dicots are grouped in two different clusters on the phylogenic tree, those from dicots are divided into several branches (Lothier *et al.*, 2011; Orsel *et al.*, 2014). Interestingly, in the Brassicaceae two different clusters group *GLN1;1*, *GLN1;2*, and *GLN1;4* as homologous genes on the one hand, and *GLN1;3* and *GLN1;5* as homologous genes in the other (Lothier *et al.*, 2011; Orsel *et al.*, 2014). Such clustering suggests similarities between the enzymes belonging to the same cluster, and our results were in good agreement with this assumption. Indeed, *GLN1;1*, *GLN1;2*, and *GLN1;4* were mainly expressed in the high-order veins while *GLN1;3* and *GLN1;5* were more specifically expressed in the low-order veins. In addition, *GLN1;1, GLN1;2*, and *GLN1;4* were over-expressed during leaf senescence and *GLN1;3* and *GLN1;5* were mainly expressed in flowers and seeds.

**Fig. 10.**
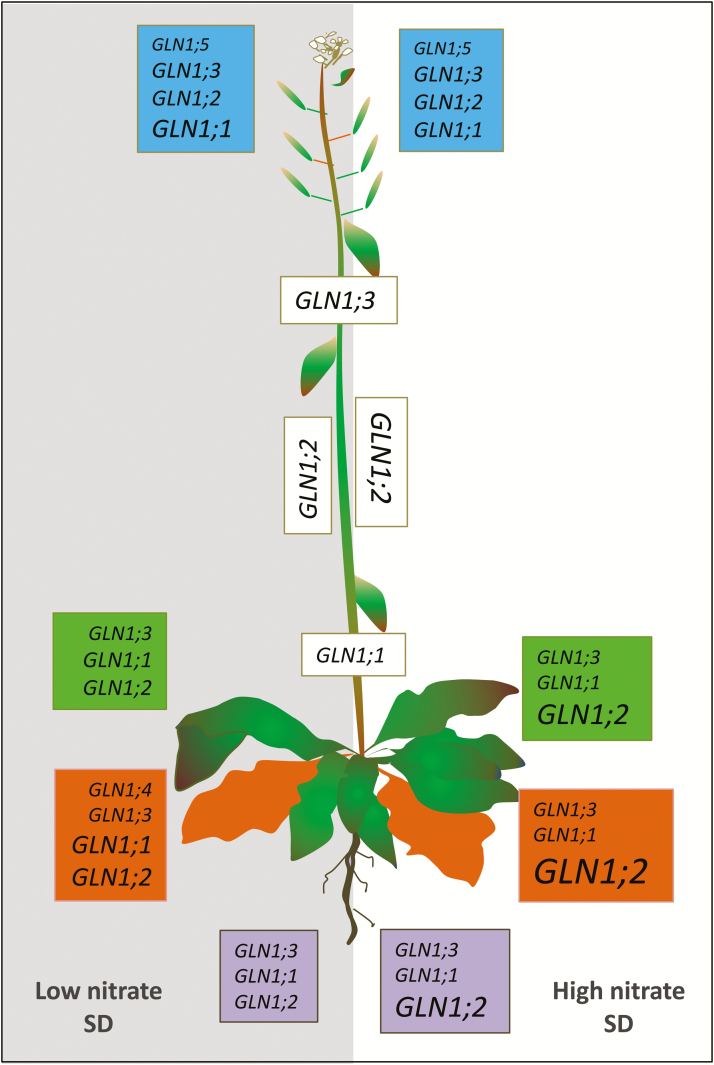
Schematic representation of the expression levels of the five *GLN1* genes in the flowers (blue), stem (white), young leaves (green), old leaves (orange), and roots (violet) of plants grown under short days (SD) and either low (left, grey background) or high (right, white background) nitrate conditions. The relative expression levels are represented by the relative size of the gene names.

Determination of the localization of *GLN1* transcriptional activities using *uidA* fusions, *GFP* fusions, and *in situ* hybridizations showed that all the five *GLN1* genes were expressed in the phloem companion cells (Supplementary Fig. S11). The expressions of *GLN1;2* and *GLN1;3* were not restricted to the companion cells and were also found in the phloem parenchyma cells. In addition, *GLN1;2* expression was detected in the mesophyll cells. The much broader expression pattern of *GLN1;2* explains its major contribution to the total GS activity in the leaf tissues, as shown in Fig. 5. The localization of the expression of the five *GLN1* genes is summarized in Fig. 10.

The total GS activity and GS1 protein content were strongly decreased in the *gln1;1-gln1;2-gln1;3* triple-mutant compared to the wild-type. Accordingly, ammonium concentrations were notably higher, and the rosettes of the triple-mutant were significantly smaller under both low- and high-nitrate conditions. In good agreement with Guan and Schjoerring (2016) and Guan *et al.*, (2015), we did not find any special phenotypes for the *gln1;1* single- and double-mutants under standard nitrate conditions. Indeed, the phenotypes for these mutants that were observed in these two previous studies were only found under hydroponic cultivation and when particular ammonium conditions were applied, but not under standard nitrate conditions. We also confirmed that under high nitrate, when ammonium biosynthesis from nitrate reduction is high, the *gln1;2* mutants (single and double) produced a smaller rosette size in the vegetative stage (Lothier *et al.*, 2011; Guan *et al.*, 2015; Konishi *et al.*, 2017).

The main purpose of our study was to determine whether the different GS1 mutants presented N-remobilization defects. Experiments using the ^15^N isotope facilitated the estimation of the major contributions of the different GS1 isoforms to N remobilization and seed filling. No differences in N remobilization or total seed yield were observed in any of the *gln1* single- and double-mutants compared to the wild-type. Only the *gln1;1-gln1;2-gln1;3* triple-mutant displayed significant defects. The decrease of N remobilization in the triple-mutant was significant under both low- and high-nitrate conditions. The lower ^15^N partitioning into the seeds of the triple-mutant was associated with retention of ^15^N in the rosettes, and also in the stems under low nitrate. As a result of its lower N-remobilization capacity, the triple-mutant produced less total seed yield and lower individual seed weight. However, the C and N composition of the seeds remained unchanged, which can be attributed to the fact that the seed sink compartment was globally reduced in the triple-mutant.

Glutamine is often described as the most abundant amino acid in the phloem sap (Tegeder and Masclaux-Daubresse, 2018). Our analyses showed that the concentrations of glutamate, aspartate, and asparagine in the phloem exudates were also important. Strikingly, the relative abundance of asparagine was much higher relative to the other amino acids in the phloem exudates than in the leaf tissues (7- to 10-fold higher). The aspartate and glutamine relative abundances were quite similar in the leaf tissues and the exudates, whilst the glutamate relative abundance was lower in the exudates than in the leaf tissues. These findings indicated that asparagine was dedicated to the phloem amino-acid flux. In plants, asparagine synthetases (ASs) catalyse the transamination of glutamine to aspartate to form glutamate and asparagine (Gaufichon *et al.*, 2010). Asparagine is also synthesized by AS through the direct condensation of ammonium on aspartate, as shown by Masclaux-Daubresse *et al.* (2006) who detected significant amounts of ^15^N-labelled asparagine in plants labelled with ^15^NH_4_^+^ and treated with the transaminase inhibitor azaserine. The higher asparagine relative abundance in the exudates of the triple-mutant and the fact that *ASN2* was significantly up-regulated in this mutant relative to the wild-type led us to deduce that nitrogen flux in the phloem of the mutant was rerouted from the conventional GS1-dependent glutamine biosynthesis to the biosynthesis of asparagine by the AS2 isoform (Gaufichon *et al.*, 2013), which is localized in the veins of Arabidopsis.

The higher levels of transcripts of *GLN2* (chloroplastic glutamine synthetase) in the triple-mutant indicated that GS2 could also compensate for the lack of glutamine biosynthesis in the mutant. The higher GS2 protein content and the higher GS2-related activity measured after ion-exchange chromatography in the triple-mutant compared to the wild-type strengthen this hypothesis. As GS2 was more abundant than GS1 in the mesophyll cells and less abundant in the phloem tissues, the increase of GS2 activity in the triple-mutant may explain the higher relative increase of glutamine percentage in the leaf tissues (+95%in the 7th leaf) than in the phloem exudates (+20%) of the mutant relative to the wild-type.

Taken together, our results showed that the triple mutation in *GLN1;1*, *GLN1;2*, and *GLN1;3* was required to produce significant effects in the nitrogen-remobilization capacity of Arabidopsis. Ammonium could be assimilated by the different GS1 isoforms located in different-order veins to provide the glutamine needed for N translocation to the seeds through the phloem. Only the *gln1;1-gln1;2-gln1;3* triple-mutation blocked efficient N remobilization, as it impaired ^15^N-ammonia re-assimilation and glutamine biosynthesis in all of the leaf vein network. We can estimate that GLN1;1, GLN1;2, and GLN1;3 together contributed to 10–20% of the total ^15^N-remobilization capacity in Arabidopsis. Such a significant but modest contribution indicated that nitrogen was certainly not only remobilized by the GS1 isoform. The sharp increase of asparagine and the higher *ASN2* transcript levels in the triple-mutant clearly indicated that asparagine synthetase also contributed to ammonium assimilation and N remobilization. The biosynthesis of glutamine in mesophyll cells by the GS2 isoform was also able to compensate for the decrease in GS1 activity. Nevertheless, the accumulation of free amino acids in the rosettes of the triple-mutant and the smaller size of the rosettes indicated that increases of GS2 and AS2 were not sufficient to restore the phenotype of *gln1;1-gln1;2-gln1;3*.

In conclusion, our study has shown that glutamine biosynthesis for N-remobilization occurs in all the high- and low-order veins in the leaves of Arabidopsis, that it is catalysed by the three major GS1 isoforms, GLN1;1, GLN1;2, and GLN1;3, and that it is alternatively supported by AS2 in the veins and GS2 in the mesophyll cells.

## Supplementary data

Supplementary data are available at *JXB* online.

Fig. S1. Characterization of the *gln1* knockout mutants.

Fig. S2. Harvest index, N allocation, and ^15^N remobilization are unchanged in any of the *gln1* single-mutants compared to the wild-type.

Fig. S3. ^15^N remobilization, seed yield, and harvest index are reduced in the *gln1;1xgln1;2xgln1;3* triple-mutant but not in the double-mutants.

Fig. S4. The *gln1;1-gln1;2-gln1;3* triple-mutant is smaller than the wild-type and other mutants.

Fig. S5. Western blots of the GS1 and GS2 isoforms in the wild-type and mutants.

Fig. S6. Cytosolic glutamine synthetase gene expression in leaves and roots under low- and high-nitrate conditions.

Fig. S7. GUS staining in flowers and stems of plants carrying transcriptional fusions of the *GLN1* promoters and the *uidA* reporter gene.

Fig. S8. Patterns of GUS activity driven by the promoters of the *GLN1* genes in transverse sections of leaf vascular tissues.

Fig. S9. Expression of the *PromGLN1*::GFP fusions in flower tissues.

Fig. S10. *In situ* hybridization of *GLN1* transcripts using specific antisense probes on transverse sections of leaf tissues.

Fig. S11. Schematic representation of the localization of the five cytosolic glutamine synthetase isoforms in the leaf vein network and their respective contribution to N remobilization in the different genetic backgrounds.

Supplementary Figures S1-S11

## Author contributions

CM-D conceived the study; MM, FS, MA, AM, SD, JL, TL HM, SC, and CM-D performed the experiments; MM, SD, MR-C, NL, and CM-D analysed, evaluated, and interpreted the data; FC was involved in discussions and statistical analyses; AM and J-CA contributed to the ^15^N analyses; CM-D supervised MM, JL, TL, and FS; CM-D co-ordinated the research and wrote the article. All the authors read and approved the final article.
