# Supplementary material for: Three cytosolic glutamine synthetase isoforms localized in different-order veins act together for N remobilization and seed filling in Arabidopsis
Source: J Exp Bot. 2018 Jun 5;69(18):4379–93. doi: 10.1093/jxb/ery217 (PMC6093384; doi:10.1093/jxb/ery217)
Supplement: Supplementary Figures S1-S11 [file ery217_suppl_supplementary_figures_s1-s11.pdf]

Supplemental information

Three cytosolic glutamine synthetase isoforms located in different order veins work together for N remobilization and seed filling in Arabidopsis.

Michael Moison[a], Anne Marmagne[a], Sylvie Dinant[a], Fabienne Soulay[a], Marianne Azzopardi[a], Jérémy Lothier[a,b,1], Sylvie Citerne [a], Halima Morin[a,2], Nicolas Legay[a,b,3], Fabien Chardon[a], Jean-Christophe Avice[c], Michèle Reisdorf-Cren[a,b] and Céline Masclaux-Daubresse[a,4]

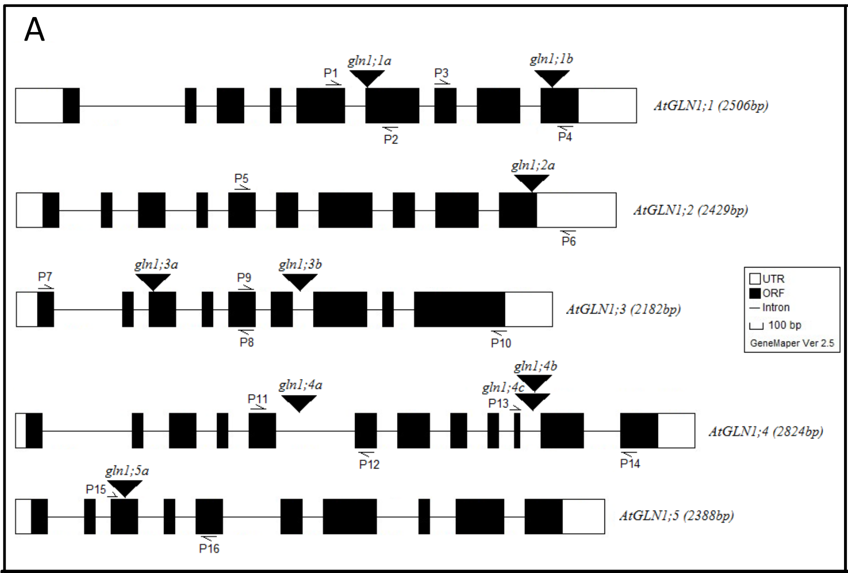

B

Line	wild-type	mutant
gln1;1a	P1/P2	P1/GABI8409
gln1;1b	P3/P4	P3/LB3
gln1;2a	P5/P6	P6/LBa1
gln1;3a	P7/P8	P7/LBa1
gln1;3b	P9/P10	P9/LBa1
gln1;4a	P11/P12	P12/LBa1
gln1;4b	P13/P14	P13/LBa1
gln1;4c	P13/P14	P13/LBa1
gln1;5a	P15/P16	P15/LBa1

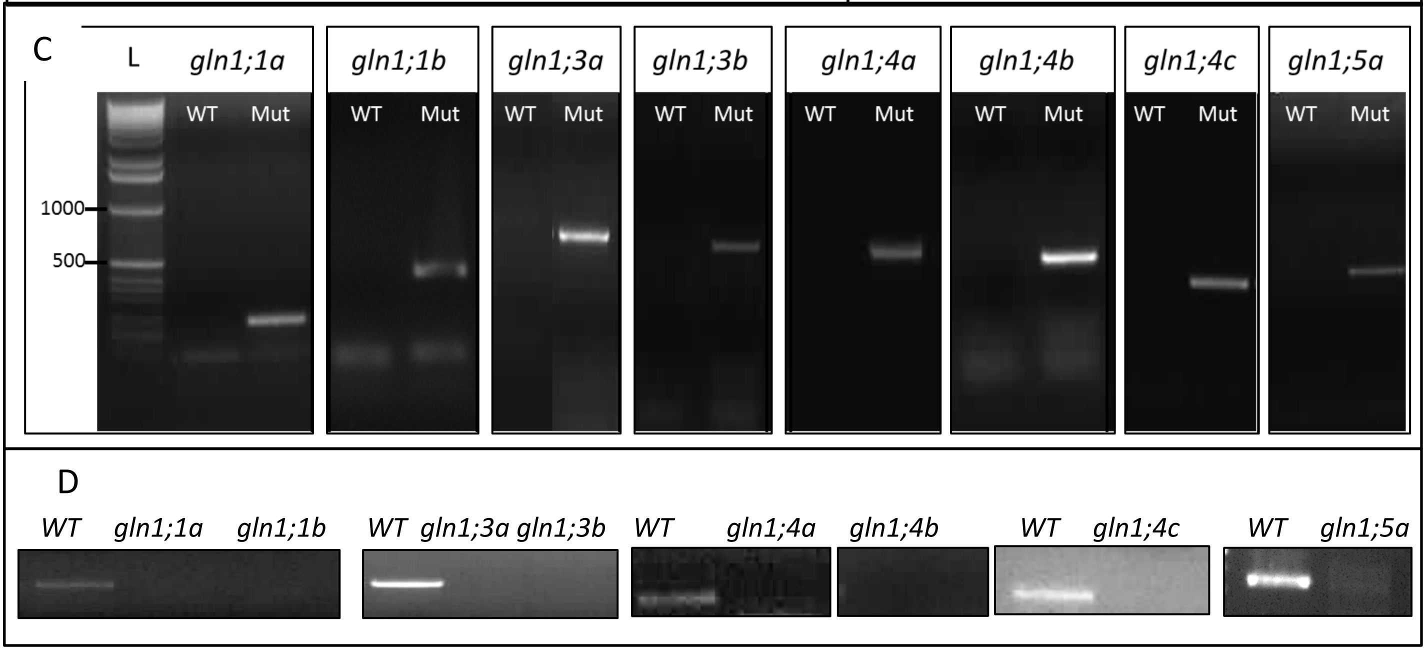

Supplemental Figure S1: *Characterization of the* gln1 *knockout mutants.* (A) Structure of Arabidopsis *GLN1* genes. Position of the T-DNA insertions are indicated by black triangles. Primers used in this study are indicated by small arrows. (B) Primers couples used for genotyping of *gln1;x* mutant line in order to detect wild-type or mutant allele; the sequence of the primers can be found in supplemental table S1. (C) Genotyping of homozygous *gln1;x* mutant lines. Amplification was only observed with primers couples aimed at amplifying mutant allele. (D) RT-PCR on mutant lines : transcripts could not be detected compared to wild-type (WT). C and D are composite images of several gels performed for mutant genotyping. Note that the *gln1;2* mutant has previously been characterized by Lothier et al. (2011).

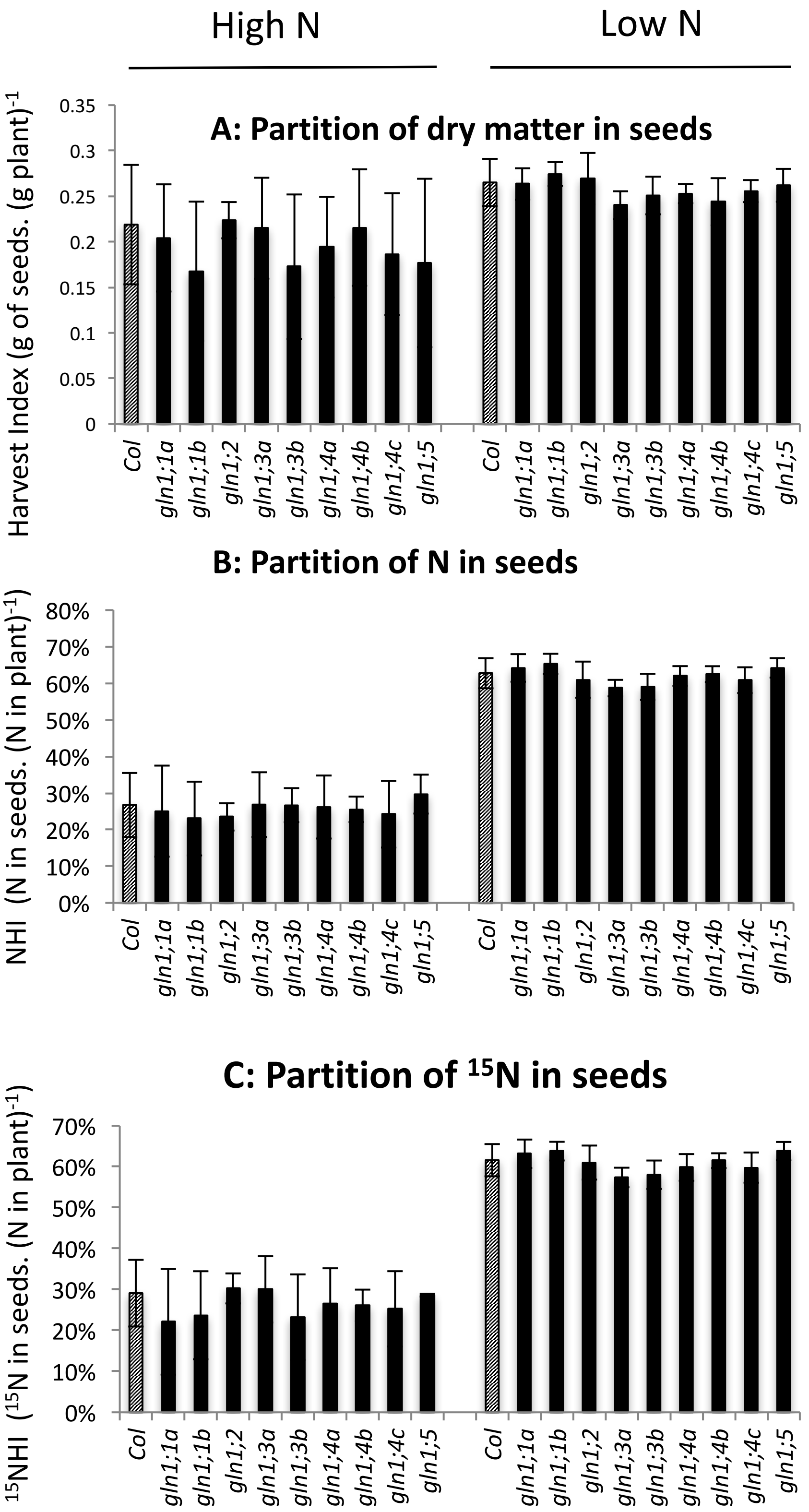

Supplemental Figure S2: ***Harvest index, N allocation and ^{15}N remobilization are unchanged in any of the* gln1 *single mutants compared to wild type*.** (A) harvest index (HI); (B) nitrogen harvest index (Partition of N in seeds, as % of the whole plant); (C) ^{15}N partition in seeds (^{15}NHI, as % of the total ^{15}N in the whole plant). Mean and standard deviation of 6 plants are shown. No significant difference between mutant and wild type could be observed.

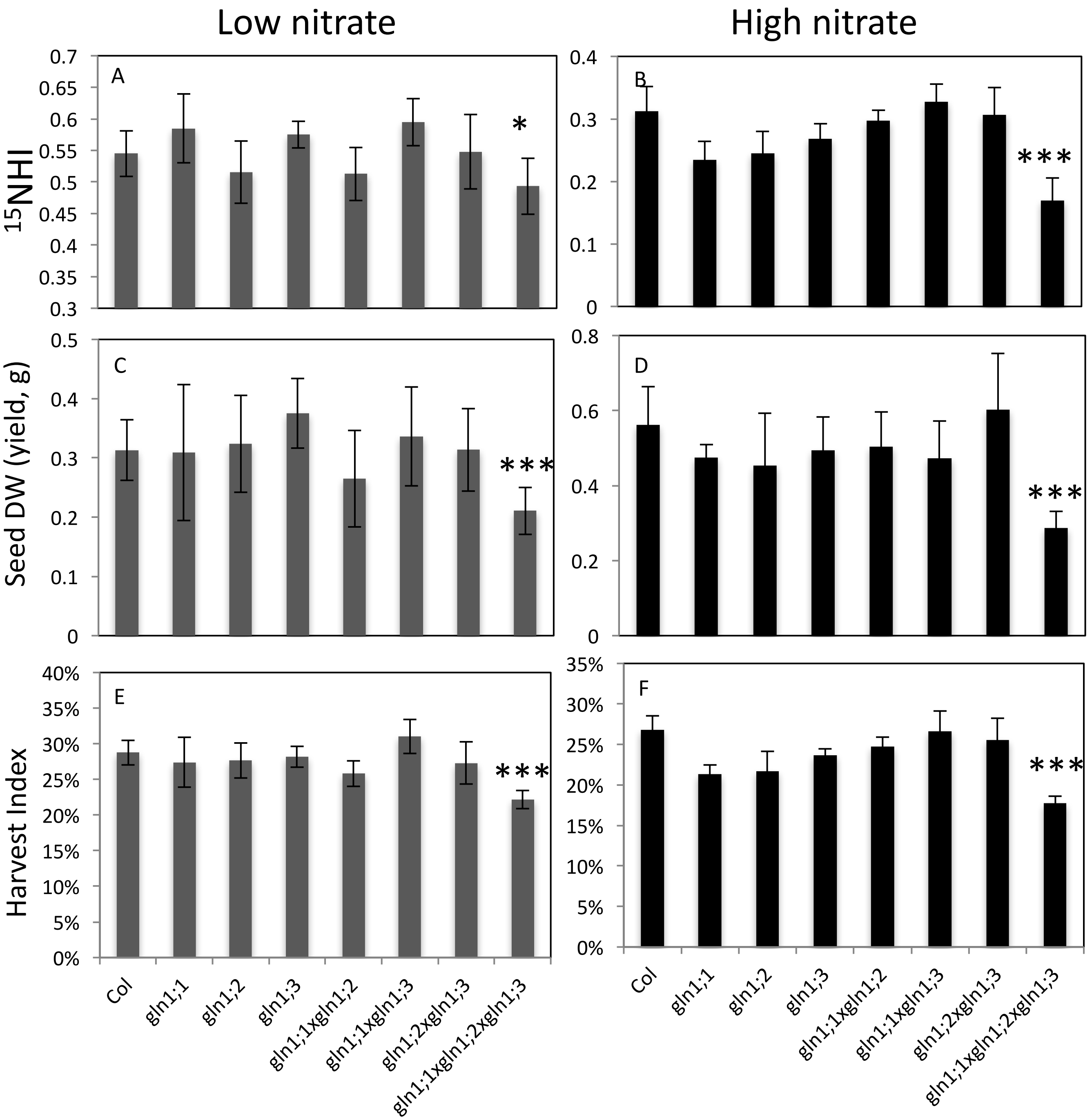

Supplemental Figure S3: ***^{15}N remobilization, seed yield and harvest index are reduced in the triple* gln1;1xgln1;2xgln1;3 *mutant but not in the double mutants.*** (A;B) ^{15}N partition in seeds (^{15}NHI, as % of the total ^{15}N in the whole plant). (C;D) Seed yield. (E;F) Harvest index. Mean and standard deviation of 6 plants are shown. Significant differences according to Student's test (* P<0.05; ** P<0.01; *** P<0.001) are indicated.

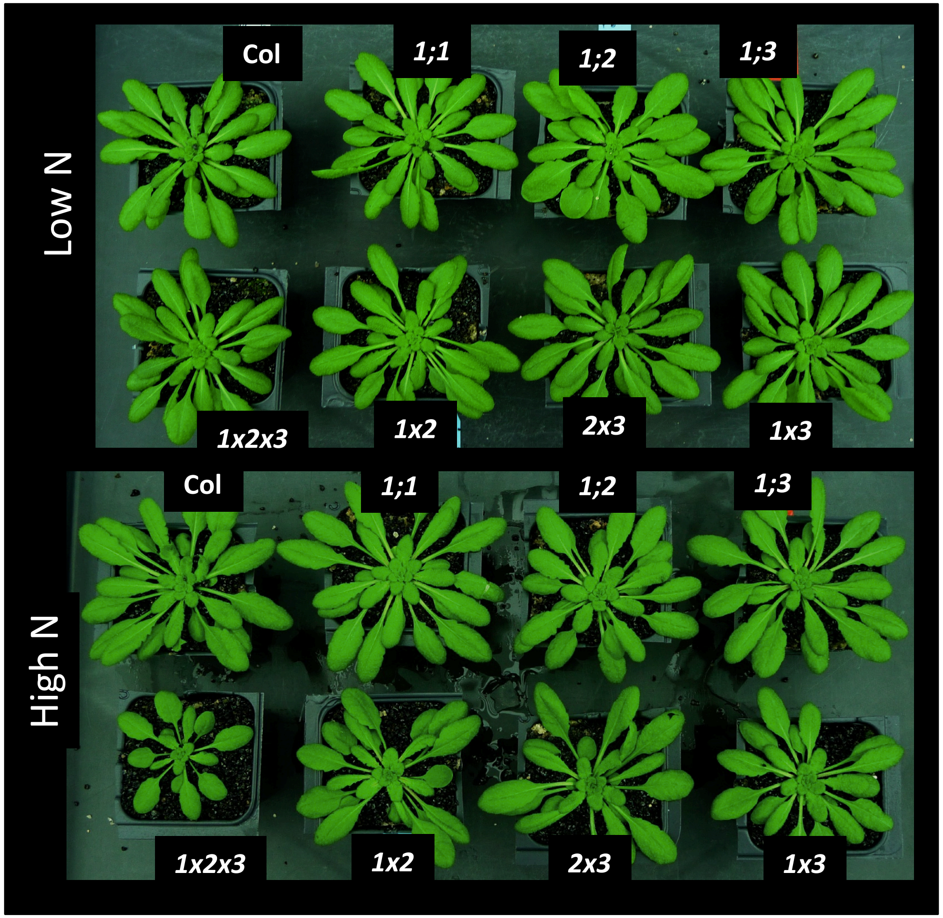

Supplemental Fig. S4: The triple gln1;1-gln1;2-gln1;3 mutant is smaller than wild type and other mutants.

The *gln1;1* (*1;1*), *gln1;2* (*1;2*), *gln1;3* (*1;3*), *gln1;1-gln1;2* (1-2), *gln1;1-gln1;3* (1x3), *gln1;2-gln1;3* (2x3) and *gln1;1-gln1;2-gln1;3* (1x2x3) mutants and wild type were growth for 45 days under short days and watered with 2 mM (Low N) or 10 mM (High N) nitrate solutions.

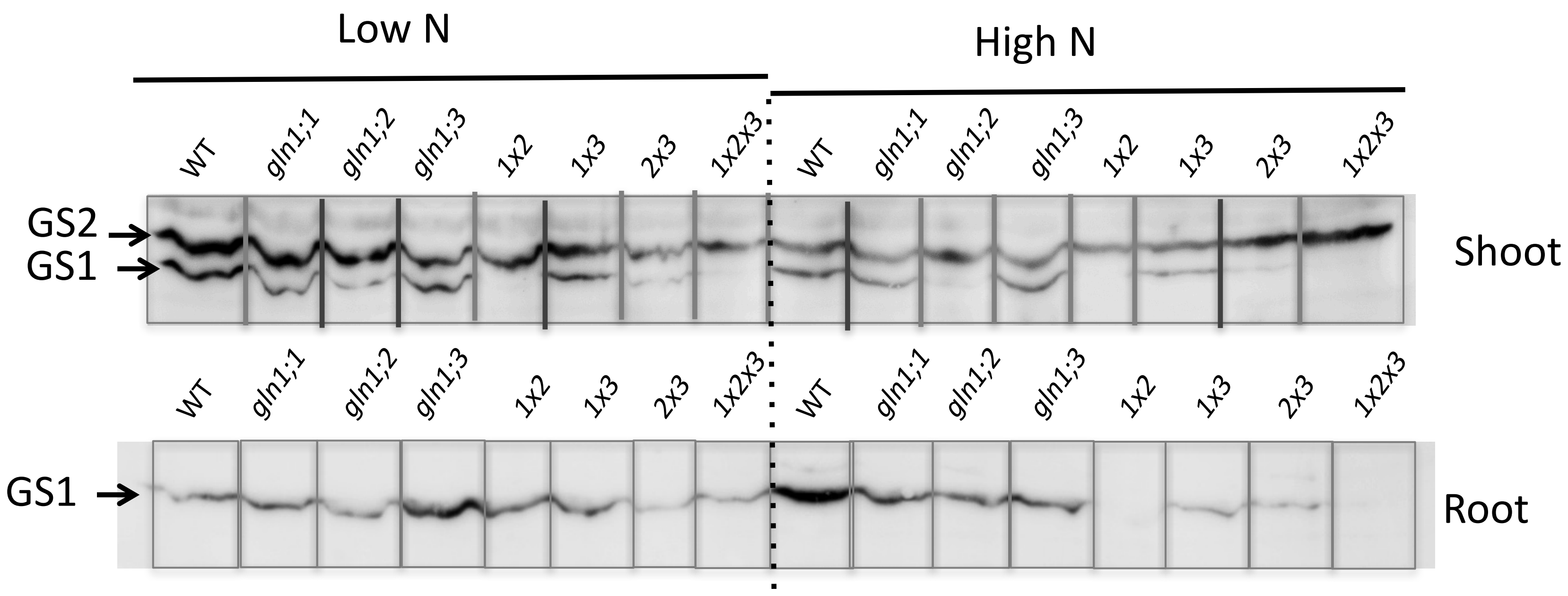

Supplemental Figure S5: ***GS1 and GS2 isoforms in wild type and mutants.*** Western blots with GS antibodies were performed on the same enzymatic extracts as used in Figure 13 in order to determine GS1 and GS2 protein levels in the roots and shoots of wild type (WT) and mutants. Western blots revealed that the GS1 protein level is strongly reduced in all the mutants carrying the *gln1;2* mutation and especially in the triple gln1;1xgln1;2xgln1;3 mutant. Equal amount of proteins (5 µg) were loaded in each lane.

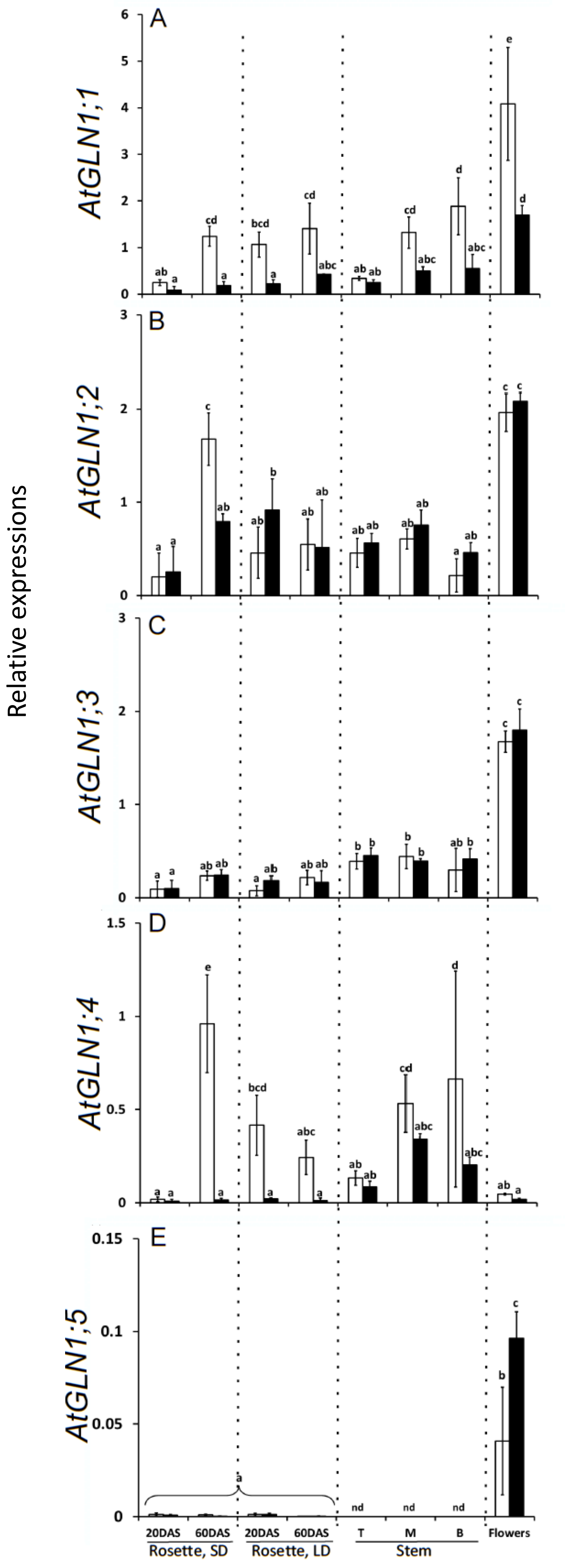

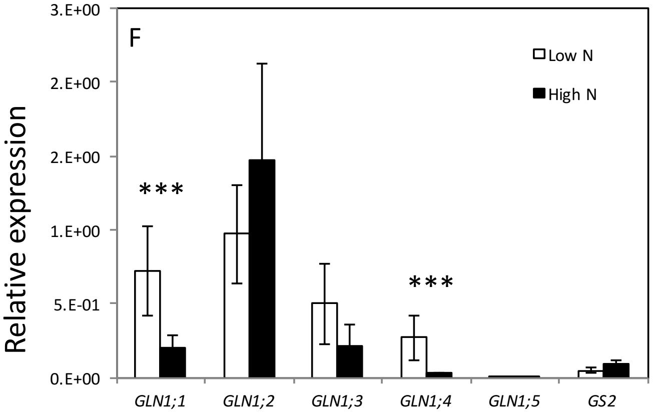

Supplemental Figure S6: ***Cytosolic glutamine synthetase gene expression in leaves and roots under low and high nitrate conditions.***

Steady-state levels of glutamine synthetase mRNA were measured using RT-qPCR in rosette (A,B,C,D) and roots (F) and are presented as relative units normalized to the $\sqrt{(Ct_{EF1\alpha4}.Ct_{APT})}$ synthetic reference gene values. Relative expression of *GLN1 and GLN2 (GS2)* are presented for plants grown under low (white bars) or high (black bars) nitrate conditions. Means and standard deviation of four biological repeats are shown. Significant differences between low and high nitrate conditions are indicated with asterisks (*** Student's test P <0.001). DAS: days after sowing; SD: short days; LD: long days; T: top; M: middle; B: bottom.

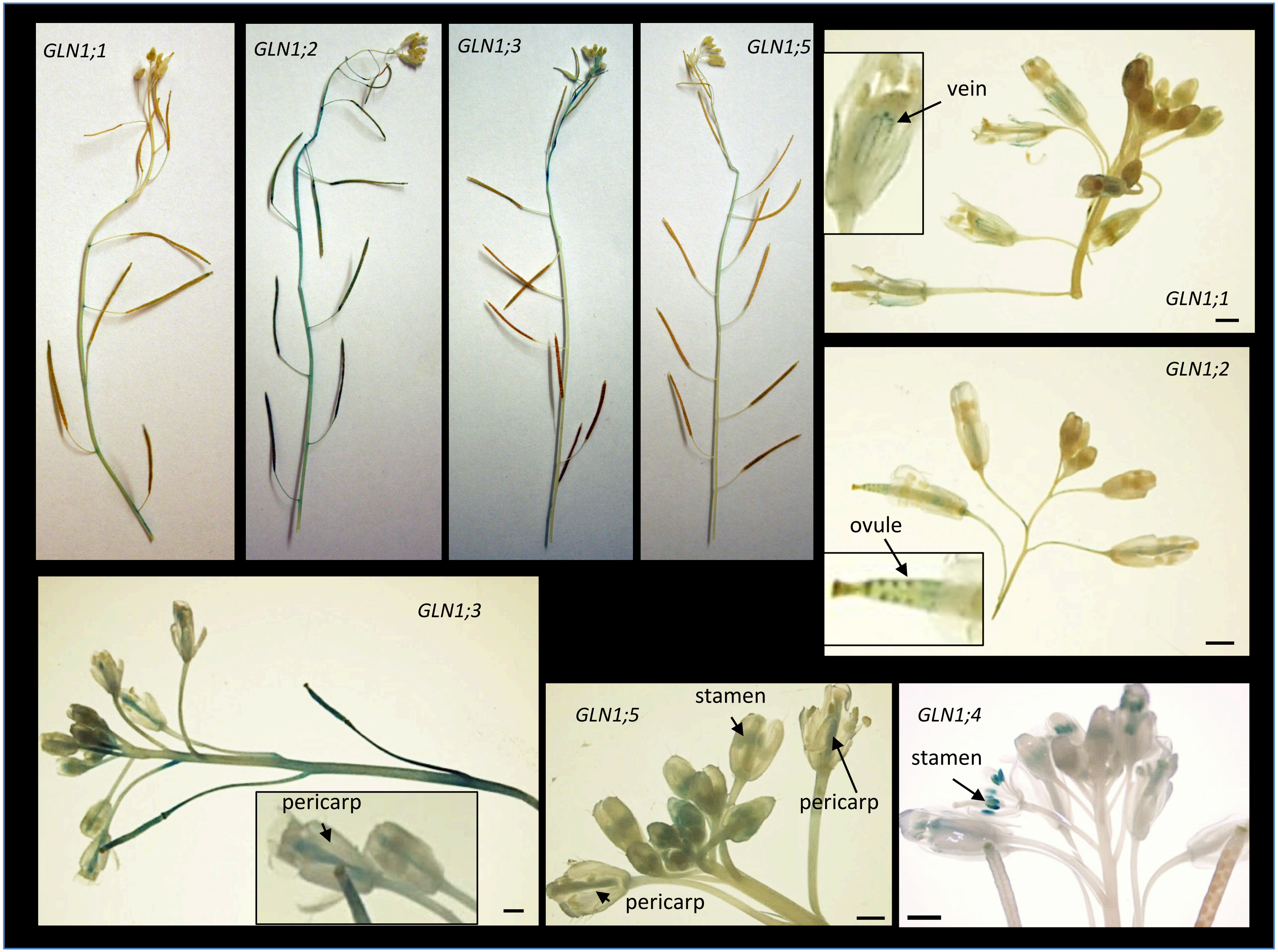

Supplemental Figure S7: ***GUS staining in flowers and stems of plants carrying transcriptional fusions of the*** **GLN1** ***promoters and*** **uidA** ***reporter gene.*** Scale bars: 1mm.

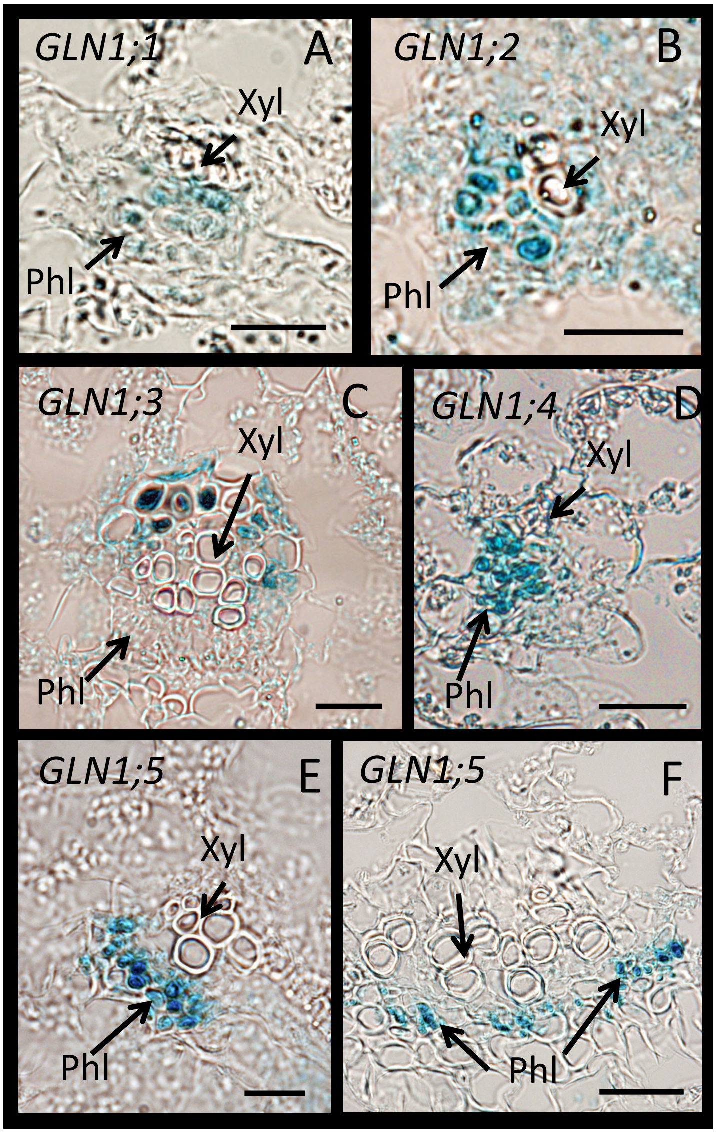

Supplemental Figure S8: Pattern of *GUS* activity driven by the promoters of the *GLN1* genes in transverse sections of leaf vascular tissues. Leaf material previously presented in Fig. 8 was embedded in agarose and cut using vibratome in order to observed transversal sections and detect the *ProGLN1;1:uidA* (A), *ProGLN1;2:uidA*. (B), *ProGLN1;3:uidA* (C), *ProGLN1;4:uidA* (D) and *ProGLN1;5:uidA* (E;F) activities. Scale bars: 20 μm. Xyl: xylem. Phl: Phloem.

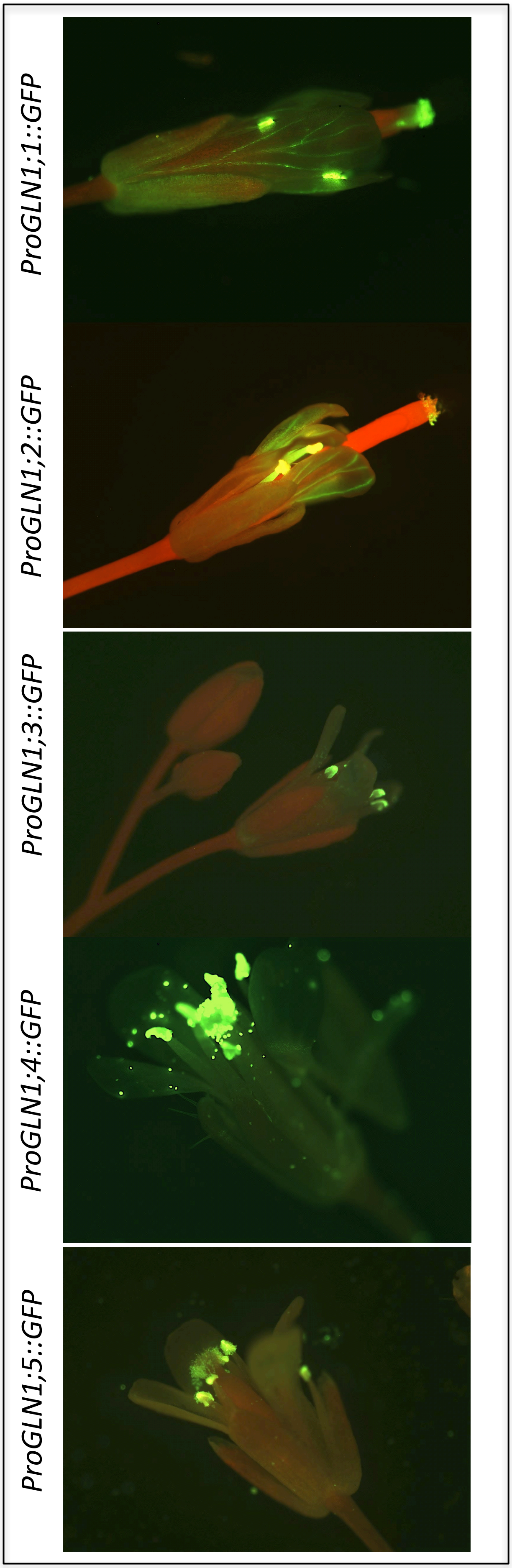

Supplemental Figure S9: ***Expression of the PromGLN1::GFP fusions in flower tissues.***

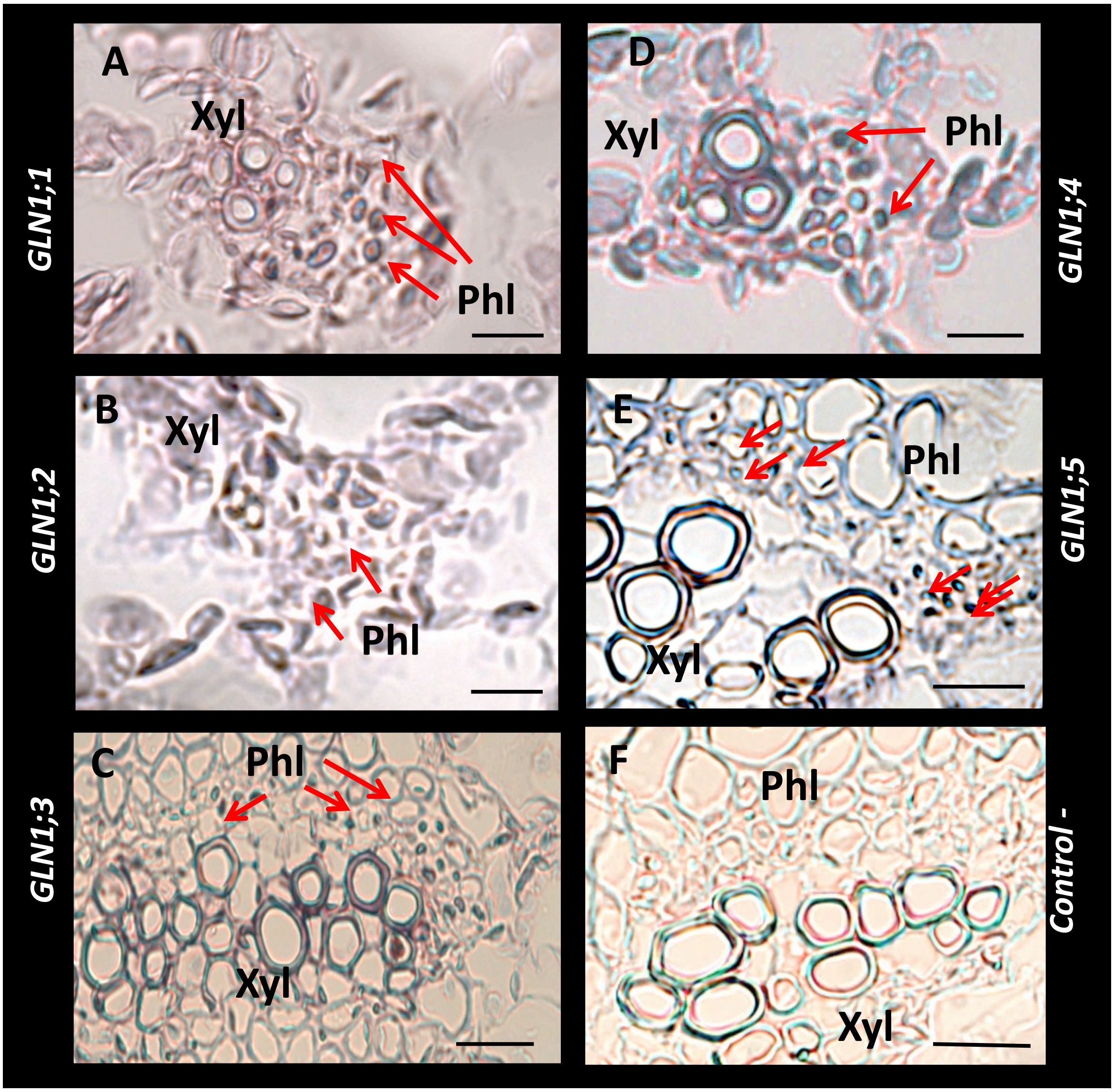

Supplemental Figure S10: In situ *hybridization of the GLN1 transcripts using specific antisens probes on transverse sections of leaf tissues.* The use of antisens probes confirmed the presence of the *GLN1;1* (A), *GLN1;2* (B), *GLN1;3* (C), *GLN1;4* (D), *GLN1;5* (E) mRNAs in small phloem cells identified as companion cells (red arrows). Negative control (F): *in situ* performed without probe. Scale bars: 10 µm. Xyl: xylem. Phl: Phloem.

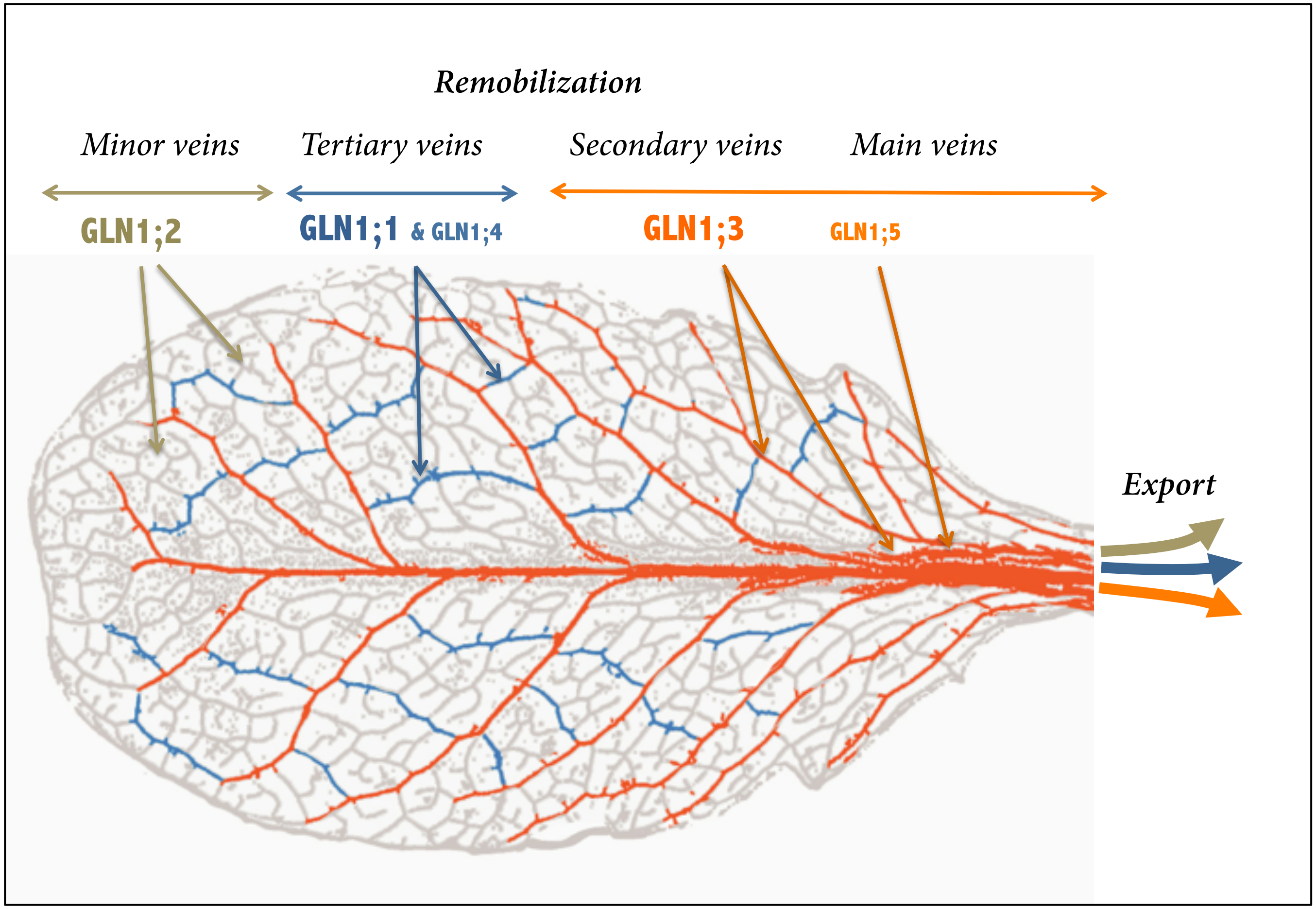

Supplemental Figure S11: Schematic representation of the localization of the five cytosolic glutamine synthetase isoforms in the leaf vein network. GLN1;2 is represented in grey, GLN1;1 and GLN1;4 are in blue, GLN1;3 and GLN1;5 are in orange.

Supplemental Table S1: T-DNA insertion mutants of *Arabidopsis thaliana* used in this study

Mutant name	Mutant line	Gène (AGI number)
gln1;1a	GK-265C09	*GLN1;1* (At5g37600)
gln1;1b	SAIL_1273_A10	
gln1;2	SALK_102291	*GLN1;2* (At1g66200)
gln1;3a	SALK_072283	*GLN1;3* (At3g17820)
gln1;3b	SALK_038156	
gln1;4a	SALK_147053	*GLN1;4* (At5g16570)
gln1;4b	SALK_039770	
gln1;4c	SALK_7138c	
gln1;5a	SALK_086579	*GLN1;5* (At1g48470)

Supplemental Table S2: List of primers used for genotyping and semiquantitative RT-PCR

Name	Gene	Sequence (5' → 3')	Use
**gln1;1a-F (P1)**	*GLN1;1* (At5g37600)	TATTGGTGGTTATCCCGGCCC	genotyping of *gln1;1a* mutant and RT-PCR
**gln1;1a-R (P2)**	*GLN1;1* (At5g37600)	TTGATCCCAGCGTATAAGCAGGCC	genotyping of *gln1;1a* mutant
**gln1;1b-F (P3)**	*GLN1;1* (At5g37600)	CACAGAGATTGCTGGTGTAGTGG	genotyping of *gln1;1b* mutant and RT-PCR
**gln1;1b-R (P4)**	*GLN1;1* (At5g37600)	TCGAAGCTGGCCTCCTGTCC	genotyping of *gln1;1b* mutant and RT-PCR
**gln1;2a-F (P5)**	*GLN1;2* (At1g66200)	GCGGGAGAGCCAATCCCTACTAACAAGCG	genotyping of *gln1;2a* mutant
**gln1;2a-R (P6)**	*GLN1;2* (At1g66200)	CCGAATTTGCTCTAACCACAGAACCC	genotyping of *gln1;2a* mutant
**gln1;3a-F (P7)**	*GLN1;3* (At3g17820)	CGTTAACCTCAACCTC	genotyping of *gln1;3a* mutant
**gln1;3a-R (P8)**	*GLN1;3* (At3g17820)	TCCTTGGCAACGTCGGGG	genotyping of *gln1;3a* mutant
**gln1;3b-F (P9)**	*GLN1;3* (At3g17820)	CAGCCACCCCGACGTTGCC	genotyping of *gln1;3b* mutant and RT-PCR on *gln1;3* mutants
**gln1;3b-R (P10)**	*GLN1;3* (At3g17820)	GTGTCATCAACCGAGTATGG	genotyping of *gln1;3b* mutant and RT-PCR on *gln1;3* mutants
**gln1;4a-F (P11)**	*GLN1;4* (At5g16570)	GTGACGCATATACACCGGC	genotyping of *gln1;4a* mutant
**gln1;4a-R (P12)**	*GLN1;4* (At5g16570)	CGACCGGCCAACCTACCGGCC	genotyping of *gln1;4a* mutant
**gln1;4b-F (P13)**	*GLN1;4* (At5g16570)	GGAGATTGGAATGGTGCAGGGGCAC	genotyping of *gln1;4b* and *gln1;4c* mutant and RT-PCR
**gln1;4b-R (P14)**	*GLN1;4* (At5g16570)	GCCCAACCCTAATCGATGCCCCACGG	genotyping of *gln1;4b* and *gln1;4c* mutant and RT-PCR
**gln1;5a-F (P15)**	*GLN1;5* (At1g48470)	CCAGGACCAGTAAGTAATCC	genotyping of *gln1;5a* mutant and RT-PCR
**gln1;5a-R (P16)**	*GLN1;5* (At1g48470)	GGAATTGGATCTCCGGCCGG	genotyping of *gln1;5a* mutant
**LB3**	T-DNA	TAGCATCTGAATTTCATAACCAATCTCGATACAC	genotyping of SAIL mutant lines
**LBa1**	T-DNA	TGGTTCACGTAGTGGGCCATCG	genotyping of SALK mutant lines
**GABI8409**	T-DNA	ATATTGACCATCATACTCATTGC	genotyping of GABI mutant lines
**RTgln1;1a-R**	*GLN1;1* (At5g37600)	TCAGCCCTTTTAAGATAATGTTTATTC	RT-PCR on *gln1;1a* mutant, reverse primer
**RTgln1;4b-F**	*GLN1;4* (At5g16570)	GAGAAGATGGAGGGTACGAGG	RT-PCR on *gln1;4b* mutant, forward primer
**RTgln1;4b-R**	*GLN1;4* (At5g16570)	GGTTTCCAAAGGATTGT	RT-PCR on *gln1;4b* mutant, reverse primer
**RTgln1;4c-F**	*GLN1;4* (At5g16570)	CAAGACCCTAGTGTTGTCGCCG	RT-PCR on *gln1;4c* mutant, forward primer
**RTgln1;5a-R**	*GLN1;5* (At1g48470)	GAATGAGACAATCACACCGC	RT-PCR on *gln1;5a* mutant, reverse primer

Table S3: List of primers used for quantitative RT-qPCR, promotor amplification and *in situ* hybridization.

Name	Gene	Sequence (5' → 3')	Use
**APT Q2+**	*APT* (At1g27450)	CGGGGATTTTAAGTGGAACA	RT-qPCR (Housekeeping gene), forward primer
**APT Q2-**	*APT* (At1g27450)	GAGACATTTTGCGTGGGATT	RT-qPCR (Housekeeping gene), reverse primer
**EF1α4up**	At5g60390	CTGGAGGTTTTGAGGCTGGTAT	
**EF1α4rp**	At5g60390	CCAAGGGTGAAAGCAAGAAGA	
**Act Q2R**	At3g18780	CCCTCGTAGATTGGCACAGT	
**ActQ1F**	At3g18780	GCCATCCAAGCTGTTCTCTC	
**qGLN1;1-F**	*GLN1;1* (At5g37600)	CAACCTTAACCTCTCAGACTCCACT	RT-qPCR, forward primer
**qGLN1;1-R**	*GLN1;1* (At5g37600)	CAGCTGCAACATCAGGGTTGCTA	RT-qPCR, reverse primer
**qGLN1;2-F**	*GLN1;2* (At1g66200)	TAACCTTGACATCTCAGACAACAGT	RT-qPCR, forward primer
**qGLN1;2-R**	*GLN1;2* (At1g66200)	TCAGCAATAACATCAGGGTTAGCA	RT-qPCR, reverse primer
**qGLN1;3-F**	*GLN1;3* (At3g17820)	TAACCTCAACCTCACCGATGCCACC	RT-qPCR, forward primer
**qGLN1;3-R**	*GLN1;3* (At3g17820)	CTTGGCAACGTCGGGGTGGCTG	RT-qPCR, reverse primer
**qGLN1;4-F**	*GLN1;4* (At5g16570)	CAATCTCGATCTCTCCGATTCCACT	RT-qPCR, forward primer
**qGLN1;4-R**	*GLN1;4* (At5g16570)	GGCGACAACACTAGGGTCTTCA	RT-qPCR, reverse primer
**qGLN1;5-F**	*GLN1;5* (At1g48470)	CCTAAACCTTGATCTATCAGACACC	RT-qPCR, forward primer
**qGLN1;5-R**	*GLN1;5* (At1g48470)	GCCTTCACATTGGGATGATCG	RT-qPCR, reverse primer
**qGLN2-F**	*GLN2* (At5g35630)	CCAACATGTCAGATGAGAGTGCC	RT-qPCR, forward primer
**qGLN2-R**	*GLN2* (At5g35630)	CCAGGTGCTTGACCGGTACTCG	RT-qPCR, reverse primer

**Prom11S**	*GLN1;1* (At5g37600)	AAAAAGCAGGCTATGGTTGTAGTCATTTCGATTGG	amplification of *GLN1;1* promoter, forward primer
**Prom11AS**	*GLN1;1* (At5g37600)	AGAAAGCTGGGTTACAAATACCAAGGGTTTGGATG	amplification of *GLN1;1* promoter, reverse primer
**Prom12S**	*GLN1;2* (At1g66200)	AAAAAGCAGGCTATTTTAGCAAGAGACCATCCACA	amplification of *GLN1;2* promoter, forward primer
**Prom12AS**	*GLN1;2* (At1g66200)	AGAAAGCTGGGTGGTTGCAAGAAGAAACAAGAAGA	amplification of *GLN1;2* promoter, reverse primer
**PGS315**	*GLN1;3* (At3g17820)	AAAAAAGCAGGCTCCTGAGCCATGCGTTGAAGGG	amplification of *GLN1;3* promoter, forward primer
**PGS313**	*GLN1;3* (At3g17820)	AAGAAAGCTGGGTCGGCGGCGTCGGAGAG	amplification of *GLN1;3* promoter, reverse primer
**PGS415**	*GLN1;4* (At5g16570)	AAAAAAGCAGGCTCTGCTTGTGGGGGACAGACC	amplification of *GLN1;4* promoter, forward primer
**PGS413**	*GLN1;4* (At5g16570)	AAGAAAGCTGGGTCCTCAAAGAATAATAAACC	amplification of *GLN1;4* promoter, reverse primer
**PGS515**	*GLN1;5* (At1g48470)	AAAAAAGCAGGCTGAAACCCTCCTTTGG	amplification of *GLN1;5* promoter, forward primer
**PGS513**	*GLN1;5* (At1g48470)	AAGAAAGCTGGGTCGGTTCTATTGATCTCAGTC	amplification of *GLN1;5* promoter, reverse primer

**HIS1.1-F**	*GLN1;1* (At5g37600)	CAATCCTCTGGAATCCTTGA	in situ hybridization, antisense probe
**HIS1.1-RT7**	*GLN1;1* (At5g37600)	TGTAATACGACTCACTATAGGGCAAAAGCAGAATAAGCAGAGCAAA	in situ hybridization, antisense probe
**HIS1.2-F**	*GLN1;2* (At1g66200)	CACTCCTCTGGAACCCTTGA	in situ hybridization, antisense probe
**HIS1.2-RT7**	*GLN1;2* (At1g66200)	TGTAATACGACTCACTATAGGGCAAAGAAAGAAAGGCCCCAAA	in situ hybridization, antisense probe
**HIS1.3-F**	*GLN1;3* (At3g17820)	CGACCATACTCGGTTGATGA	in situ hybridization, antisense probe
**HIS1.3-RT7**	*GLN1;3* (At3g17820)	TGTAATACGACTCACTATAGGGCTGTCACAACAATACGCCCATA	in situ hybridization, antisense probe
**HIS1.4-F**	*GLN1;4* (At5g16570)	TCCACAATCCTTTGGAAACC	in situ hybridization, antisense probe
**HIS1.4-RT7**	*GLN1;4* (At5g16570)	TGTAATACGACTCACTATAGGGCTCGTTCTCAGAAACAAATTGAAGA	in situ hybridization, antisense probe
**HIS1.5-F**	*GLN1;5* (At1g48470)	TGCTGAAACCACCATCCTCT	in situ hybridization, antisense probe
**HIS1.5-RT7**	*GLN1;5* (At1g48470)	TGTAATACGACTCACTATAGGGCTGAAAGAAAAGTCTAAATGCGAAA	in situ hybridization, antisense probe

Three cytosolic glutamine synthetase isoforms located in different order veins work together for N remobilization and seed filling in Arabidopsis.

Michael Moison[a], Anne Marmagne[a], Sylvie Dinant[a], Fabienne Soulay[a], Marianne Azzopardi[a], Jérémy Lothier[a,b,1], Sylvie Citerne [a], Halima Morin[a,2], Nicolas Legay[a,b,3], Fabien Chardon[a], Jean-Christophe Avice[c], Michèle Reisdorf-Cren[a,b] and Céline Masclaux-Daubresse[a,4]

Table S4: relative proportion of individual amino acids in phloem exudates, 7th leaf and rosette of wild type (Col) and triple mutant (1x2x3) under low (2 mM) and high (10 mM) nitrate conditions. Mean and SD of 3-4 biological replicate are shown. Significant differences between WT and triple mutant are in italic and tagged in yellow.

	Phloem exudates		7th leaf		Rosette	
**2mM**	**Col**	**1x2x3**	**Col**	**1x2x3**	**Col**	**1x2x3**
**ASP**	19.7 ± 2.2	19.8 ± 5.7	24.7 ± 1.7	25.1 ± 1.3	20.4 ± 1.0	20.1 ± 0.8
**THR**	8.1 ± 1.6	7.3 ± 1.3	3.7 ± 0.4	3.7 ± 0.1	3.0 ± 0.1	3.2 ± 0.1
**SER**	6.9 ± 1.8	7.4 ± 1.7	8.4 ± 1.6	8.8 ± 1.9	4.6 ± 0.3	5.1 ± 0.5
**ASN**	10.0 ± 0.9	13.0 ± 4.2	***1.3 ± 0.1***	***2.2 ± 0.3***	***3.3 ± 0.3***	***5.6 ± 0.2***
**GLU**	23.1 ± 3.6	19.6 ± 3.5	***39.3 ± 1.3***	***32.5 ± 1.2***	***35.7 ± 1.3***	***28.9 ± 0.4***
**GLN**	25.6 ± 6.0	27.0 ± 3.4	***11.5 ± 0.9***	***18.2 ± 1.5***	***23.4 ± 1.5***	***27.8 ± 0.9***
**ALA**	6.4 ± 1.1	6.5 ± 1.4	4.4 ± 0.4	4.1 ± 0.7	3.8 ± 0.4	3.5 ± 0.1
**VAL**	3.8 ± 2.0	4.1 ± 0.9	1.4 ± 0.3	1.3 ± 0.1	2.2 ± 0.1	2.1 ± 0.1
**ILE**	nd	nd	0.3 ± 0.3	0.5 ± 0.3	0.3 ± 0.1	0.3 ± 0.1
**LEU**	nd	nd	***0.3 ± 0.1***	***0.4 ± 0.1***	0.3 ± 0.0	0.4 ± 0.1
**PHE**	1.8 ± 0.3	1.4 ± 0.4	0.2 ± 0.1	0.2 ± 0.1	0.3 ± 0.2	0.3 ± 0.0
**LYS**	4.2 ± 0.7	3.5 ± 0.7	0.4 ± 0.1	0.3 ± 0.2	0.3 ± 0.1	0.2 ± 0.0
**ARG**	2.9 ± 0.9	3.2 ± 1.2	0.6 ± 0.1	0.6 ± 0.1	***0.5 ± 0.0***	***0.7 ± 0.1***
**GLY**	nd	nd	2.3 ± 2.7	0.6 ± 0.5	0.7 ± 0.4	0.6 ± 0.1
**TYR**	nd	nd	0.1 ± 0.1	0.2 ± 0.1	0.1 ± 0.1	0.1 ± 0.0
**HIS**	nd	nd	0.4 ± 0.3	0.5 ± 0.1	0.3 ± 0.1	0.4 ± 0.0
**PRO**	nd	nd	0.9 ± 0.5	0.6 ± 0.2	0.7 ± 0.0	0.8 ± 0.3
**10mM**	**Col**	**1x2x3**	**Col**	**1x2x3**	**Col**	**1x2x3**
**ASP**	17.0 ± 1.0	16.0 ± 2.0	25.8 ± 2.0	25.0 ± 1.4	21.6 ± 0.3	21.5 ± 0.8
**THR**	6.0 ± 0.0	6.0 ± 1.0	5.2 ± 1.1	4.7 ± 1.8	3.4 ± 0.2	3.3 ± 0.2
**SER**	6.0 ± 1.0	6.0 ± 1.0	6.2 ± 1.3	6.0 ± 2.2	3.6 ± 0.3	3.6 ± 0.3
**ASN**	***9.0 ± 1.0***	***14.0 ± 2.0***	***1.4 ± 0.1***	***2.7 ± 0.7***	***3.9 ± 0.2***	***5.6 ± 0.2***
**GLU**	18.0 ± 1.0	18.0 ± 3.0	***36.7 ± 2.9***	***30.7 ± 2.0***	***32.5 ± 0.8***	***29.8 ± 1.0***
**GLN**	***18.0 ± 2.0***	***22.0 ± 2.0***	***10.9 ± 3.7***	***21.3 ± 1.8***	25.7 ± 0.6	26.5 ± 0.8
**ALA**	5.0 ± 0.0	6.0 ± 1.0	4.8 ± 0.7	4.4 ± 1.4	3.7 ± 0.6	3.6 ± 0.4
**VAL**	4.0 ± 0.0	3.0 ± 1.0	1.1 ± 0.6	1.2 ± 1.5	2.0 ± 0.2	2.1 ± 0.2
**ILE**	2.0 ± 1.0	2.0 ± 1.0	0.7 ± 0.4	0.9 ± 0.7	0.3 ± 0.1	0.2 ± 0.1
**LEU**	2.0 ± 1.0	2.0 ± 1.0	0.8 ± 0.6	0.8 ± 0.8	0.3 ± 0.1	0.2 ± 0.1
**PHE**	1.0 ± 0.0	1.0 ± 1.0	0.1 ± 0.1	0.2 ± 0.3	0.2 ± 0.1	0.2 ± 0.0
**LYS**	***2.0 ± 0.0***	***3.0 ± 0.0***	0.5 ± 0.2	0.6 ± 0.4	0.3 ± 0.0	0.2 ± 0.0
**ARG**	3.0 ± 0.0	3.0 ± 1.0	0.8 ± 0.5	0.6 ± 0.5	0.6 ± 0.1	0.5 ± 0.0
**GLY**	1.0 ± 0.0	1.0 ± 1.0	1.1 ± 0.8	1.0 ± 0.4	0.5 ± 0.1	0.8 ± 0.1
**TYR**	1.0 ± 0.0	1.0 ± 1.0	0.6 ± 0.9	0.5 ± 0.6	0.1 ± 0.0	0.1 ± 0.0
**HIS**	1.0 ± 1.0	1.0 ± 1.0	0.6 ± 0.7	0.9 ± 0.4	0.4 ± 0.0	0.4 ± 0.1
**PRO**	3.0 ± 2.0	3.0 ± 2.0	1.1 ± 1.1	1.0 ± 0.8	1.1 ± 0.3	1.3 ± 0.1
